# Short-term dynamics of fecal microbiome and antibiotic resistance in juvenile rainbow trout (*Oncorhynchus mykiss*) following antibiotic treatment and withdrawal

**DOI:** 10.1186/s42523-024-00361-0

**Published:** 2024-12-20

**Authors:** Min Kyo Kim, Yoonhang Lee, Jiyeon Park, Ju-Yeop Lee, Hyo-Young Kang, Young-Ung Heo, Do-Hyung Kim

**Affiliations:** 1 Department of Aquatic Life Medicine, Pukyong National University, Busan, Republic of Korea; 2 Microbial Oceanography Laboratory, School of Earth and Environmental Sciences and Research Institute of Oceanography, Seoul National University, Seoul, 08826 Republic of Korea; 3 School of Fisheries, Aquaculture and Aquatic Sciences, Auburn University, Auburn, AL 36849 USA

**Keywords:** Antimicrobial resistance, Dysbiosis, Fecal microbiome, Non-invasive sampling, Rainbow trout (*Oncorhynchus mykiss*), Restoration

## Abstract

**Background:**

In aquaculture, the secretions of cultured organisms contribute to the development of aquatic antibiotic resistance. However, the antibiotic-induced changes in fish feces remain poorly understood. This study aimed to assess the short-term dynamics of fecal microbiome and antibiotic resistance in juvenile rainbow trout (*Oncorhynchus mykiss*) upon antibiotic treatment and withdrawal period.

**Methods:**

Fish were orally administered diets supplemented with oxytetracycline (OTC) or sulfadiazine/trimethoprim (SDZ/TMP) for 10 consecutive days, followed by a 25-day withdrawal period. Fecal samples were collected before antibiotic treatment (day 0), and at 1, 3, 7, and 10 days post antibiotic administration (dpa), as well as 1, 3, 7, 14, and 25 days post antibiotic cessation (dpc). The fecal microbiome community was profiled using both culture-dependent and -independent methods. The relative abundance of antibiotic resistance genes (ARGs) and the class 1 integron-integrase gene (*intI1*) in the feces were quantified using real-time PCR.

**Results:**

Antibiotic treatment disrupted the fecal microbial communities, and this alteration persisted even after antibiotic cessation. Moreover, OTC treatment increased the relative abundance of *tet* genes, while *sul* and *dfr* genes increased in the SDZ/TMP-treated group. Notably, *Flavobacterium*, *Pseudomonas*, and *Streptococcus* exhibited a significant correlation with the abundance of ARGs, suggesting their potential role as carriers for ARGs.

**Conclusion:**

This study demonstrates the antibiotic-induced changes in the fecal microbiome and the increase of ARGs in rainbow trout feces. These findings provide novel insights into the dynamics of microbiome recovery post-antibiotic cessation and suggest that fish feces provide a non-invasive approach to predict changes in the fish gut microbiome and resistome.

**Supplementary Information:**

The online version contains supplementary material available at 10.1186/s42523-024-00361-0.

## Introduction

Antibiotic resistance is a global public health concern. Antibiotics are used as an important tool in the treatment of human and animal disease [1], but excessive use of antibiotics can promote the emergence of antibiotic resistant bacteria (ARB) and the spread of antibiotic resistance genes (ARG) [2]. This phenomenon is fueling the spread of antibiotic resistance worldwide due to close contact and resource sharing between humans and animals [1]. In particular, with the high diversity and intensity of bacteria, aquaculture is vulnerable to the introduction and proliferation of ARBs and ARGs [3]. Aquaculture systems are highly complex, dynamic, interconnected, and easily influenced by environmental and anthropogenic factors [4]. Antibiotics are sometimes administered preventively in aquaculture, even in the absence of disease, which increases the risk of resistance development and antibiotic residues in aquatic environments [1, 5]. Also, due to their low bioavailability, many antibiotics are poorly absorbed, with approximately 25–75% excreted into the environment via feces or urine [6, 7]. Consequently, unabsorbed antibiotics and secretions of culture organisms enter aquatic environments close to aquaculture facilities and provide a favorable environment for the enrichment of persistent aquatic ARGs [8]. Therefore, as aquaculture has become a hotspot for the emergence and dissemination of ARBs and ARGs [9], several studies on the impacts of antibiotic treatment on fish and aquatic environments have been extensively conducted.

Antibiotic treatment in fish can disrupt the healthy gut microbial community, leading to dysbiosis [10, 11]. Persistent dysbiosis can lead to deficiencies in the host immune system [12], making it crucial to restore the imbalanced microbial community to its normal state. Extensive research on this topic has been conducted in various vertebrate animals, including broilers and humans. These studies have reported varying recovery durations, ranging from 12 days to more than 4 years after antibiotic withdrawal [13]. In a recent study on catfish (*Silurus meridionalis*) administered with florfenicol, the microbial community was found to recover to a normal state within 7 days after the cessation of treatment [14]. In addition, another study showed a complete restoration of the gut microbiome within 10–15 days after the withdrawal of florfenicol treatment in snubnose pompano (*Trachinotus blochii*) [15]. However, in hybrid grouper (*Epinephelus fuscoguttatus* ♀ × *E. lanceolatus* ♂) treated with oxytetracycline, no recovery was observed even after two weeks of treatment withdrawal [16]. This highlights the complexity and variability of microbial community recovery in different fish species.

Rainbow trout is one of the worldwide freshwater farmed fish species, accounting for 1.3% (744,000 tons) of total aquaculture production in 2021, with a production of 2,483 tons in Korea in the same year [17, 18]. Given its significance in aquaculture, rainbow trout has been the subject of numerous studies on microbial community changes due to antibiotic treatment [19, 20]. However, most of these studies have focused on microbial shifts during the antibiotic treatment period, resulting in less attention on the persistence of antibiotic resistance and the recovery of microbial communities after treatment cessation. Understanding how microbial communities in rainbow trout respond to antibiotic treatment and how they recover after treatment cessation is crucial for better management of antibiotic use in aquaculture.

Additionally, previous studies have predominantly focused on analyzing the changes in microbial communities within the fish gut following antibiotic administration. However, the sampling methods using intestine samples require sacrificing animals, prompting recent investigations to adopt non-intrusive and non-contact strategies using fecal samples [21]. The non-invasive sampling method eliminates the need to harm or sacrifice animals, minimizing distress and ensuring laboratory welfare in animal trials [22]. In addition, this method allows for repeated sampling from the same animal over time, making it ideal for tracking the continuity and temporal dynamics of the gut microbiome and monitoring the different life stages of a particular animal [23]. Fecal sampling has been found suitable for detecting antibiotic resistance in the gut microbiome in chicken [21], and fecal samples have shown similarities to the large intestinal microbiota in lizards [24]. Furthermore, fecal sampling has proven to be an accurate assessment tool for studying the large intestinal microbiota in birds [25]. Despite these studies on other species, limited research has been conducted on the impacts of antibiotic pressure specifically on fish feces.

Therefore, in this study, fecal samples of rainbow trout treated with oxytetracycline (OTC) and sulfadiazine/trimethoprim (SDZ/TMP), which are widely used in aquaculture, were used [20, 26]. The changes in the microbial community before, during, and after antibiotic treatment were investigated by culture-dependent and -independent methods. ARGs and *intI1* were quantitatively analyzed through real-time PCR, and the correlation between ARGs, *intI1*, and the microbiome was analyzed. The objectives of this study were to examine the dynamics in the fecal microbiome and the emergence of antibiotic resistance following antibiotic treatment and to investigate the persistence of antibiotic resistance and microbial dysbiosis following treatment cessation. Additionally, this study aimed to assess the potential of fish feces as a non-invasive approach to monitor microbiome recovery and antibiotic resistance in aquaculture settings.

## Materials and methods

### Experimental design

The animal experiment was approved by the Ethics Committee of Pukyong National University (approval number: PKNUIACUC-2023-28) and conducted according to the Bioethics and Safety Act of the South Korean Ministry of Health and Welfare. Clinically healthy rainbow trout (*Oncorhynchus mykiss*) were purchased from a fish farm in Sangju, Korea. Fifteen fish with an average weight of 20 ± 3.4 g, were randomly distributed among three tanks and acclimated at 15 ^o^C for 10 days. During this period, the fish were fed with commercial non-medicated dry pellets (DongA One, Korea), once per day at a rate of 2.5% of body weight. Commercial dry pellets consist of crude protein (52%), crude ash (15%), crude fat (10%), phosphorus (2.7%), crude fiber (2%), and calcium (1.2%). Following a 10-day acclimation period, five fish per tank were fed diets surface-coated with oxytetracycline hydrochloride (Sigma-Aldrich, USA) or sulfadiazine/trimethoprim (Sigma-Aldrich, USA) following the standard protocol [27]. OTC was administrated at 75 mg/kg of body weight/day and SDZ/TMP at 30 mg/kg of body weight/day. OTC or SDZ/TMP was adsorbed into the feed pellets through hand mixing for 5 min, and the diets were administered by oral route at a rate of 2.5% of body weight for 10 consecutive days. The remaining tank served as a control group, and the fish received only non-medicated feed mixed with 0.85% saline throughout the experiment. After the treatment period, all fish received the non-medicated feed by oral route at a rate of 2.5% of body weight for 25 days. Throughout the entire period, the feeding response of individual fish was monitored to ensure the complete diet allocation was consumed. In addition, regular measurements of weight and length were conducted every five days for all fish in each group.

### Culture-dependent analysis of fecal bacterial community

**Fig. 1:**
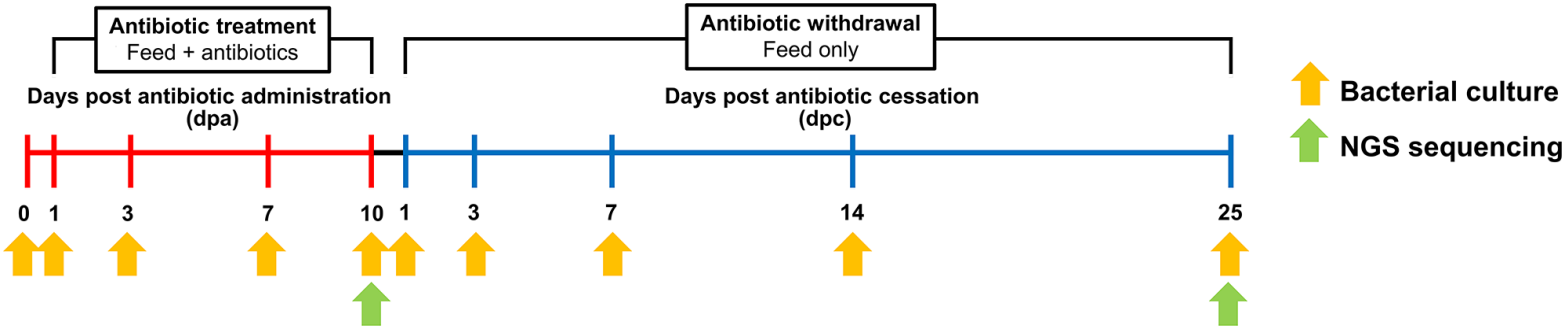
Schematic plan illustrating the treatment and withdrawal period. Rainbow trout were fed diets supplemented with oxytetracycline (OTC) or sulfadiazine/trimethoprim (SDZ/TMP) for 10 consecutive days. Following the 10-day treatment period, all fish were provided non-medicated feed for 25 days. Fecal samples were collected at 10 different time points during the experiment

Fish feces were collected from each tank at ten different time points with three replicates: immediately before antibiotic treatment (day 0), and at 1, 3, 7, and 10 days post-antibiotic administration (dpa), as well as 1, 3, 7, 14, and 25 days post antibiotic cessation (dpc) (Fig. 1). Throughout the entire period, 100% of the water was exchanged daily, 24 h after fish feeding. At each sampling time point, fish feces were collected from each tank using the siphoning method during water exchange. The collected samples were centrifuged at 8,000 rpm for 3 min at 15 ^o^C to remove residual water, and the wet weight of the fecal samples was measured. Fecal samples (100 mg wet weight) from each tank were homogenized using tissuelyser (Qiagen, Germany) and vigorously vortexed in 1 ml of sterile saline (0.85% NaCl). Serial dilutions of the homogenized fecal samples were spread onto tryptone soya agar (TSA, Oxoid, UK) supplemented with 0.3% yeast extract (TSAY) plates and incubated at 25 ^o^C for up to 7 days. Equivalent amounts of feces and homogenized fecal samples from each tank were stored at -20 ^o^C until further use.

The total bacterial colonies were enumerated, and individual colonies were selected based on colony morphology for bacterial identification. All colonies were pure cultured on fresh media, and they were stored at -70 ^o^C in tryptone soya broth (TSB; Oxoid, UK) supplemented with 10% (v/v) glycerol. Genomic DNA were extracted using AccuPrep Genomic DNA Extraction kit (Bioneer, Korea). For bacterial identification, bacterial 16S rRNA gene was amplified using universal primer sets: 27F (5’-AGA-GTT-TGA-TCM-TGG-CTC-AG-3′) and 1492R (5′-TAC-GGY-TAC-CTT-GTT-ACG-ACT-T-3′) and HS Prime Taq Premix (GeNetBio, Korea) following the PCR condition in Lane [28]. PCR products were purified using an AccuPrep PCR/Gel Purification kit (Bioneer, Korea), and sequenced using 3730XL DNA Analyzer (ThermoFisher, USA). Sequences were taxonomically assigned with Greengenes database. To enhance taxonomic resolution, strains that were either unclassified at the species level or exhibited less than 97% similarity in partial 16S rRNA gene sequences [29] were selected. These strains were subsequently compared with the closest type strain identified through Basic Local Alignment Search Tool (BLAST) analysis.

### Culture-independent analysis using the Illumina iSeq system

DNA was extracted from frozen fecal samples (10 dpa and 25 dpc) using the QIAamp DNA Stool Mini Kit (Qiagen, Germany) following the manufacturer’s instructions. The quality and concentration of extracted DNA were assessed using Qubit 3.0 Fluorometer with the Qubit™ dsDNA HS assay (Promega, USA), and NanoVue Plus Spectrophotometer (GE Healthcare, USA), respectively. The purified DNA was stored at -20 ^o^C until further analysis.

Sequencing libraries were prepared by a two-step PCR amplification. In the amplicon PCR, the 16S rRNA gene was amplified using 2X KAPA HiFi HotStart Ready Mix (KAPA Biosystems, USA), template DNA, and 16S V4 primers with Illumina adaptor overhang sequences (515F: 5′-TCG-TCG-GCA-GCG-TCA-GAT-GTG-TAT-AAG-AGA-CAG-GTG-CCA-GCM-GCC-GCG-GTA-A-3′, 806R: 5′-GTC-TCG-TGG-GCT-CGG-AGA-TGT-GTA-TAA-GAG-ACA-GGA-CTA-CHV-GGG-TAT-CTA-ATC-C-3′). The PCR products were purified using AMPure XP beads (Beckman Coulter Genomics, USA), and indexing PCR amplification was carried out according to the manufacturer’s guidelines (Nextera XT Index Kit, Illumina, USA). AMPure XP beads were used for indexed PCR amplicon clean-up, and the final amplicon concentration was quantified using the Qubit 3.0 Fluorometer with the Qubit™ dsDNA HS assay (Promega, USA). The purified libraries were pooled in equimolar concentrations. After adding a 10% PhiX Control library (Illumina, USA), the library was loaded onto an iSeq-100 reagent cartridge (Illumina, USA) and sequenced on an iSeq-100 platform (Illumina, USA) using the iSeq reagent Kits v2 (2 × 150 bp; 300 cycles) to generate 2 × 150 bp paired-end sequence reads.

### Quantification of antibiotic resistance genes

Total DNA was extracted from 100 mg of each homogenized fecal sample, with three replicates per sample, using AccuPrep Stool DNA Extraction kit (Bioneer, Korea) following the manufacturer’s instructions. The concentration and purity of extracted DNA were analyzed using a NanoVue Plus Spectrophotometer, and the purified DNA was stored at -20 ^o^C until further analysis.

Before conducting quantitative analysis of ARGs, the presence of individual ARGs, which are known to be frequently detected in freshwater [30], and farmed fish [31, 32], was initially confirmed using conventional PCR. The conventional PCR reactions were performed using 34 primer sets (20 *tet* genes, four *sul* genes, and ten *dfr* genes). Detailed information about the primer is summarized (Supplementary table S1). Subsequently, sequencing was conducted on some PCR products to validate the accuracy of the results, and the genes confirmed to be present based on the conventional PCR results were subjected to quantitative analysis. For the quantification of antibiotic resistance genes and mobile genetic element (MGE) abundances, real-time PCR (qPCR) assay was performed using the Agilent AriaMX Real-time PCR system (Agilent, USA). The qPCR reactions were performed using 17 primer sets (Supplementary table S2). Among these, 15 sets targeted ARGs associated with three primary antibiotic classes (tetracycline, sulfonamide, and trimethoprim). The remaining sets were used for MGE linked to class 1 integrons, and the 16S rRNA gene served as a reference gene for normalization of ARGs and MGE abundance. The qPCR was carried out using AccuPower 2X Greenstar qPCR Master Mix (Bioneer, Korea) according to the manufacturer’s protocol. The relative abundance of ARGs and MGE to total bacteria in each DNA sample was calculated using a relative quantification method, which has been employed in several studies to calculate the relative abundance of ARGs [33, 34]. This method normalized the abundance of the target genes to the 16S rRNA control gene and expressed as Relative abundance (RA) [35]:

$$\:\Delta{\text{C}}_{\text{T}}\:=\:{\text{C}}_{\text{T}}\:\left(\text{T}\text{a}\text{r}\text{g}\text{e}\text{t}\:\text{g}\text{e}\text{n}\text{e}\right)\:-\:{\text{C}}_{\text{T}}\:\left(16\text{S}\:\text{r}\text{R}\text{N}\text{A}\:\text{g}\text{e}\text{n}\text{e}\right)$$

$$\:\Delta\Delta{\text{C}}_{\text{T}}\:=\:\Delta{\text{C}}_{\text{T}}\:(\text{A}\text{n}\text{t}\text{i}\text{b}\text{i}\text{o}\text{t}\text{i}\text{c}-\text{t}\text{r}\text{e}\text{a}\text{t}\text{e}\text{d}\:\text{s}\text{a}\text{m}\text{p}\text{l}\text{e})\:-\:\Delta{\text{C}}_{\text{T}}\:\left(\text{U}\text{n}\text{t}\text{r}\text{e}\text{a}\text{t}\text{e}\text{d}\:\text{s}\text{a}\text{m}\text{p}\text{l}\text{e}\right)$$

$$\:\text{R}\text{A}\:\left(\text{R}\text{e}\text{l}\text{a}\text{t}\text{i}\text{v}\text{e}\:\text{a}\text{b}\text{u}\text{n}\text{d}\text{a}\text{n}\text{c}\text{e}\right)\hspace{0.17em}=\hspace{0.17em}{2}^{-\Delta\Delta{\text{C}}_{\text{T}}}$$

### Bioinformatic analysis

After Illumina iSeq sequencing, the characteristics of raw sequences were analyzed using FastQC (Version 0.12.1), and low-quality sequences (Q-score < 20) and adapters were trimmed using Trimmomatic (Version 0.38). *De novo* clustering was performed to identify operational taxonomic units (OTUs) with 97% sequence similarity using QIIME2 [36]. The taxonomic representations of all OTUs were annotated using the Greengenes database [37]. Suspected contamination was detected in one of the samples collected from the SDZ/TMP group on 10 dpa, so that sample was excluded from subsequent analysis.

Most of the analyses including alpha diversity, beta diversity, taxonomic abundance, and linear discriminant analysis of effect size (LEfSe) were calculated and visualized using the MicrobiomeAnalyst (Version 2.0) [38]. Data were normalized by the trimmed mean of M-values. Alpha diversity between control and antibiotic-treated groups was measured by Observed OTUs, Chao1 richness (microbial richness), Inverse Simpson (microbial diversity), and the Shannon Diversity indices (microbial evenness). In addition, beta diversity in microbiome community composition among groups was analyzed using the Bray-Curtis dissimilarity matrix [39]. The Bray-Curtis dissimilarity matrix was then visualized using principal coordinate analysis (PCoA) and non-metric multidimensional scaling (NMDS) methods. Furthermore, LEfSe analysis was performed to determine whether any OTUs were differentially abundant between control and antibiotic-treated groups, respectively. The effect size of each bacterial taxon with differential abundance was estimated using Linear discriminant analysis (LDA). LDA scores can be interpreted as the degree of consistent difference in relative abundance between control and antibiotic-treated groups, and the threshold on the logarithmic score of LDA analysis was set to 2.0 [40].

### Correlation analysis and validation

The relative abundance of ARGs and *intI1* from qPCR results was used to calculate Pearson’s correlation coefficients between ARGs and *intI1* using R software (Version 4.2.3). In addition, based on the relative abundance of ARGs, *intI1*, and microbial abundance data from 10 dpa and 25 dpc, Pearson’s correlation coefficient was used to assess the correlations between microbiome, *intI1*, and ARGs. Association network among ARGs, *intI1*, and fecal microbiome visualized in Cytoscape (Version 3.9.1). To validate the reliability of the correlation analysis between ARGs and the microbial community, a BLAST analysis was conducted using the Nucleotide collection (nt) database in the National Center for Biotechnology Information (NCBI). The nucleotide sequences of the 15 ARGs tested in this study were obtained in FASTA format from the Comprehensive Antibiotic Resistance Database. The BLAST searches were conducted using the acquired ARG sequences as queries. For each BLAST search, the “Search Set” was customized to include only organisms belonging to the specific genus (Supplementary table S3). The BLAST parameters were configured to ensure stringent alignment criteria and appropriate filtering to exclude low-quality matches. The BLAST search results were then verified to determine whether the identified genus possessed the corresponding ARGs.

### Statistical analysis

Length and weight of five fish per group, viable bacterial counts, the relative abundance of cultured bacterial phyla, and relative abundance of ARGs and *intI1* from three replicates per group are presented as mean ± standard deviation (SD). Statistical analysis was performed using one-way analysis of variance (ANOVA) with post-hoc Duncan multiple comparisons to determine significant differences among the control, OTC, and SDZ/TMP groups (*p*-value ≤ 0.05). Additionally, alpha diversity from three replicates per group was tested using Welch’s ANOVA with post-hoc Duncan multiple comparisons, while beta diversity from three replicates per group was analyzed using PERMANOVA with post-hoc pairwise PERMANOVA [41] to compare differences among groups. For LEfSe analysis, the Kruskal-Wallis H test with post-hoc Wilcoxon rank test was used to evaluate differences between three replicates from control and antibiotic-treated groups. Furthermore, the statistical significance of correlations was assessed with *p*-value less than 0.05.

## Results

### Fish performance

All the experimental fish consumed the provided feed completely, and there was no abnormal behavior, clinical symptoms, and mortality of fish during the experiment. Additionally, the average length and weight of the fish did not show a statistically significant difference among the control, OTC-, and SDZ/TMP-treated groups (Supplementary table S4; Supplementary table S5).

### Enumeration and composition of cultured bacteria

**Fig. 2:**
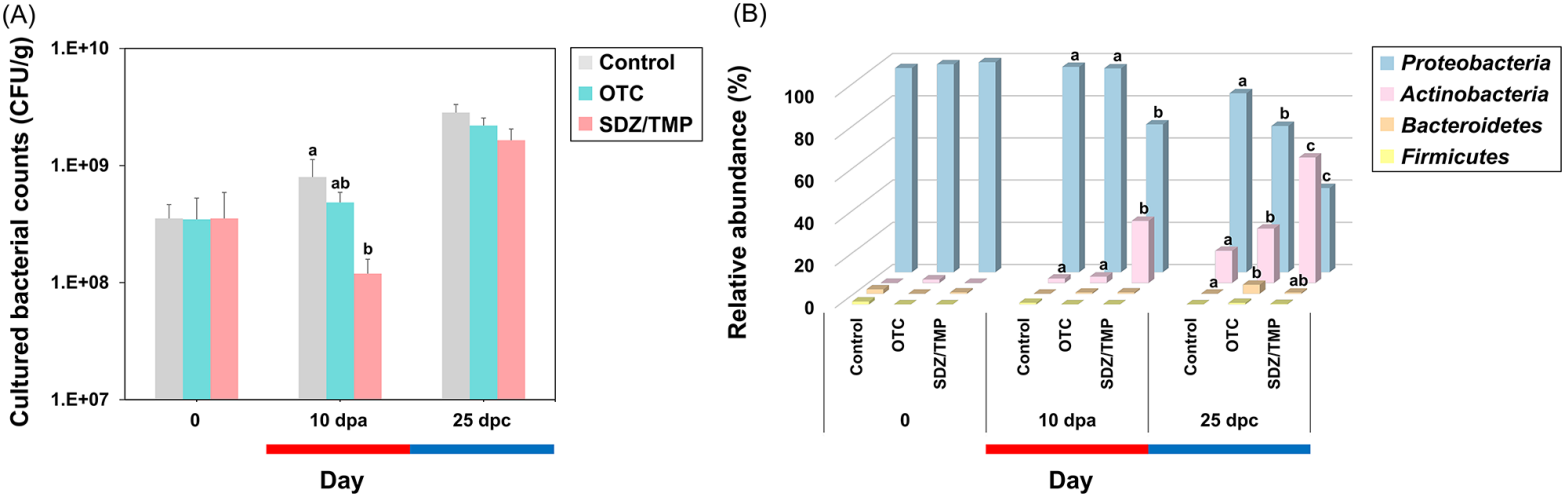
Changes in counts (**A**) and relative abundance (**B**) of cultured bacteria in rainbow trout feces. Feces from control, oxytetracycline (OTC)-, and sulfadiazine/trimethoprim (SDZ/TMP)-treated rainbow trout were analyzed. Abbreviations “dpa” and “dpc” represent “days post antibiotic administration” and “days post antibiotic cessation”, respectively. Results for 0 day, 10 dpa, and 25 dpc are shown. Statistically significant differences among groups were assessed using a one-way ANOVA followed by Duncan’s multiple range test

The average number of cultured bacteria on TSAY plates from rainbow trout feces ranged from 10^8^ to 10^9^ CFU/g (Fig. 2A; Supplementary figure S1; Supplementary table S6). At day 0, there were no significant differences in bacterial counts between the groups. By day 10, bacterial counts decreased significantly in both the OTC-treated group and SDZ/TMP-treated group compared to the control group. However, after 7 dpc, no significant differences in bacterial counts were observed between the control group and both antibiotic-treated groups.

A total of 33 bacterial species were identified from rainbow trout feces. During the antibiotic treatment period, several species exhibited significant alterations in their presence compared to the control group (Fig. 2B; Supplementary figure S2; Supplementary table S7). At the phylum level, the fecal microbiome composition in the SDZ/TMP-treated group shifted significantly, with increased *Actinobacteria* and decreased *Proteobacteria* at 3 and 10 dpa (Fig. 2B; Supplementary table S8; Supplementary table S9). Additionally, on the last day of the withdrawal period (25 dpc), both the antibiotic-treated groups had significantly less *Proteobacteria* and more *Actinobacteria* than the control group.

### Microbiome diversity through Illumina iSeq sequencing

A total of 3,008,091 sequence reads of 16S rRNA gene were generated, with varying sequence lengths (Supplementary figure S3). The effective sequences were clustered into 327 OTUs at a 97% similarity threshold. Rarefaction curves and Good’s coverage index confirmed that sequencing efforts were sufficient to capture the complete diversity of fecal bacterial communities (Supplementary figure S4; Supplementary table S10).

**Fig. 3:**
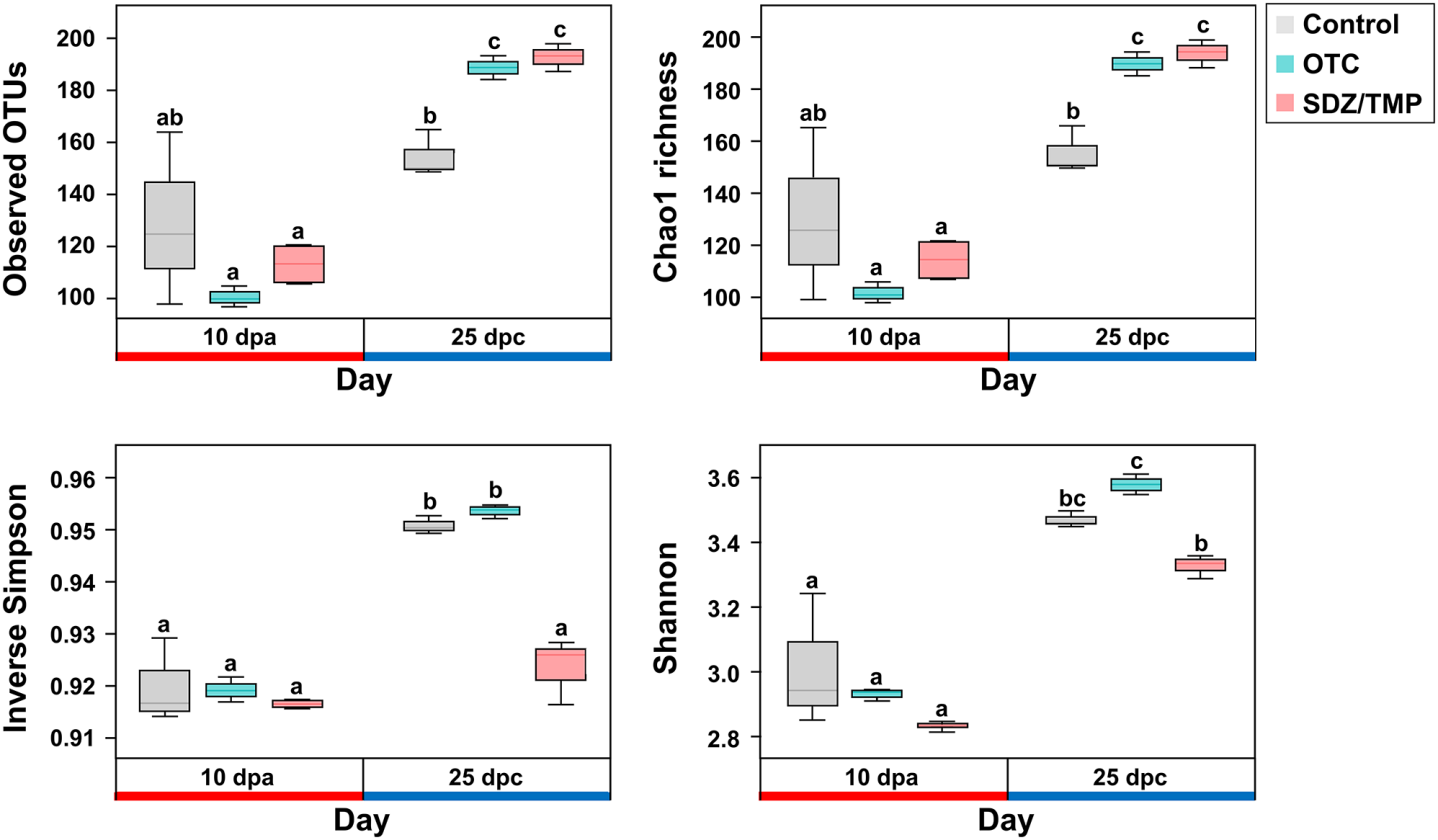
Alpha diversity (Observed OTUs, Chao1, Inverse Simpson, Shannon index) of rainbow trout fecal microbiome communities. These measures were assessed in the feces of control, oxytetracycline (OTC)-, and sulfadiazine/trimethoprim (SDZ/TMP)-treated rainbow trout at 10 dpa and 25 dpc. Error bars indicate the 95% confidence interval; the top, middle, and bottom of each box represent the 75th, 50th, and 25th percentiles, respectively. Groups with different letters were significantly different (*p*-value ≤ 0.05) based on Welch’s ANOVA test

Alpha-diversity analysis of the fecal microbial communities revealed significant changes among the groups (Fig. 3). Antibiotic treatment increased the number of observed OTUs at 25 dpc compared to 10 dpa in all groups. The antibiotic-treated groups (OTC, 188.67 ± 4.51; SDZ/TMP, 193.33 ± 5.03) showed a significantly higher number of observed OTUs compared to the control group (154.67 ± 8.96) at 25 dpc. Similar patterns were observed in the Chao1 index, indicating higher microbial richness in the antibiotic-treated groups (OTC, 189.33 ± 4.62; SDZ/TMP, 193.53 ± 5.27) compared to the control group (155.12 ± 9.14) at 25 dpc. Conversely, the inverse Simpson index demonstrated that microbial evenness was significantly lower in the SDZ/TMP-treated group (0.92 ± 0.01) than in the control (0.95 ± 0.00) and OTC-treated groups (0.95 ± 0.00) at 25 dpc. Similarly, the Shannon index indicated that microbial richness and evenness were higher in the OTC-treated group (3.57 ± 0.03) compared to the SDZ/TMP-treated group (3.32 ± 0.03) at 25 dpc.

**Fig. 4:**
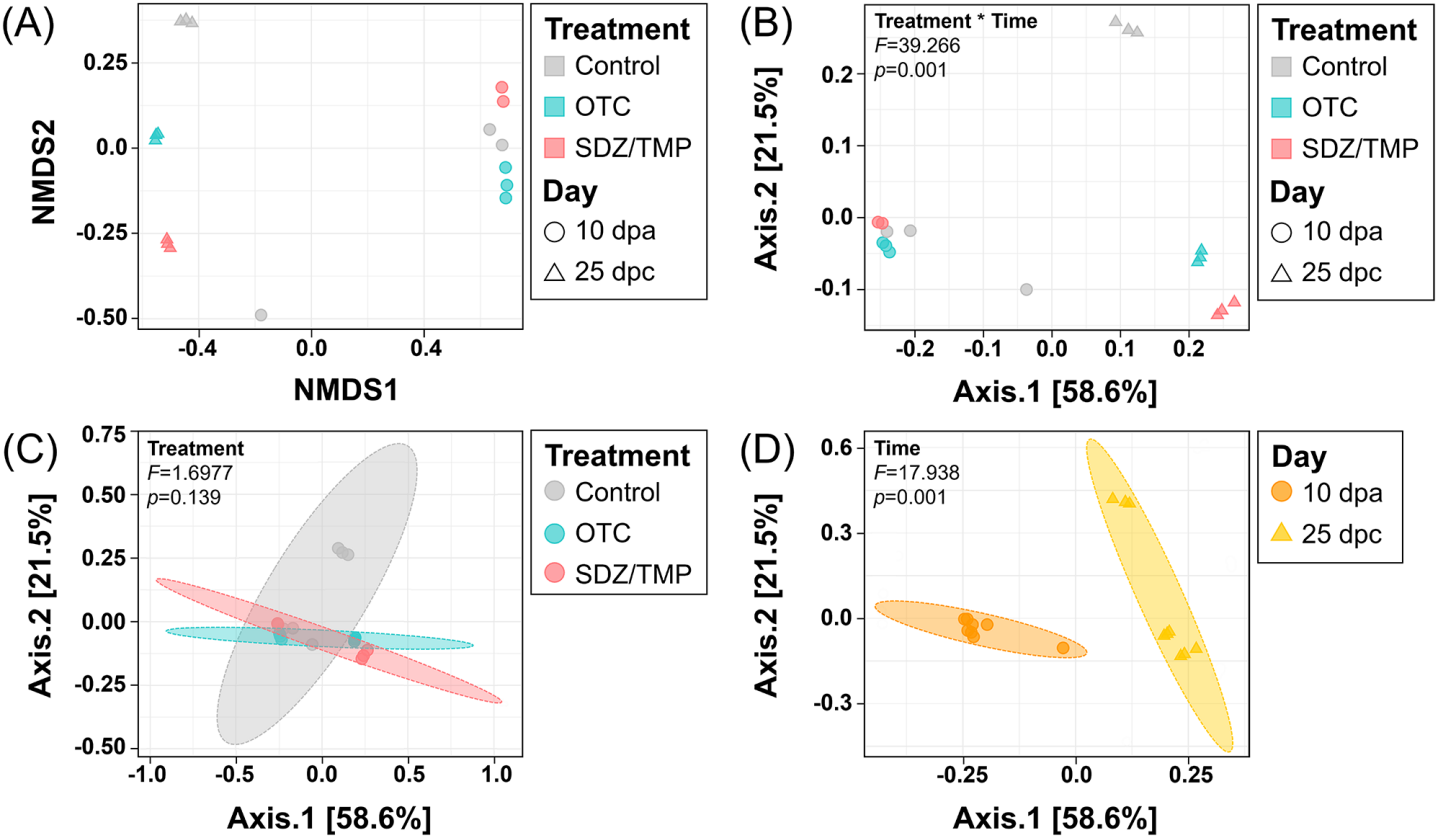
Beta diversity according to treatment-by-time (**A**, **B**), treatment (**C**), and time (**D**) in rainbow trout. Feces from control, oxytetracycline (OTC)-, and sulfadiazine/trimethoprim (SDZ/TMP)-treated rainbow trout at 10 dpa and 25 dpc were analyzed. Non-metric multidimensional scaling; NMDS (A) and Principal coordinate analysis; PCoA (B-D) were illustrated the dissimilarity indices calculated using the Bray-Curtis dissimilarity matrix between the bacterial communities. The PERMANOVA *F* and *p*-value are provided for each plot

Beta diversity analysis of rainbow trout fecal microbiomes was visualized through non-metric multidimensional scaling and principal coordinate analysis for all groups (Fig. 4). The results demonstrated that the fecal microbiomes of rainbow trout clustered significantly when both time and antibiotic treatment factors were considered (PERMANOVA; *F* = 39.27; Fig. 4B). When time was analyzed as the sole factor, clustering was significant but with a reduced *F*-value (PERMANOVA; *F* = 17.94; Fig. 4D). In contrast, no significant differences in fecal microbiomes were observed among the control, OTC-, and SDZ/TMP-treated groups, when treatment was considered solely (PERMANOVA; *p* = 0.14) (Fig. 4C).

### Microbiome community based on Illumina iSeq sequencing

**Fig. 5:**
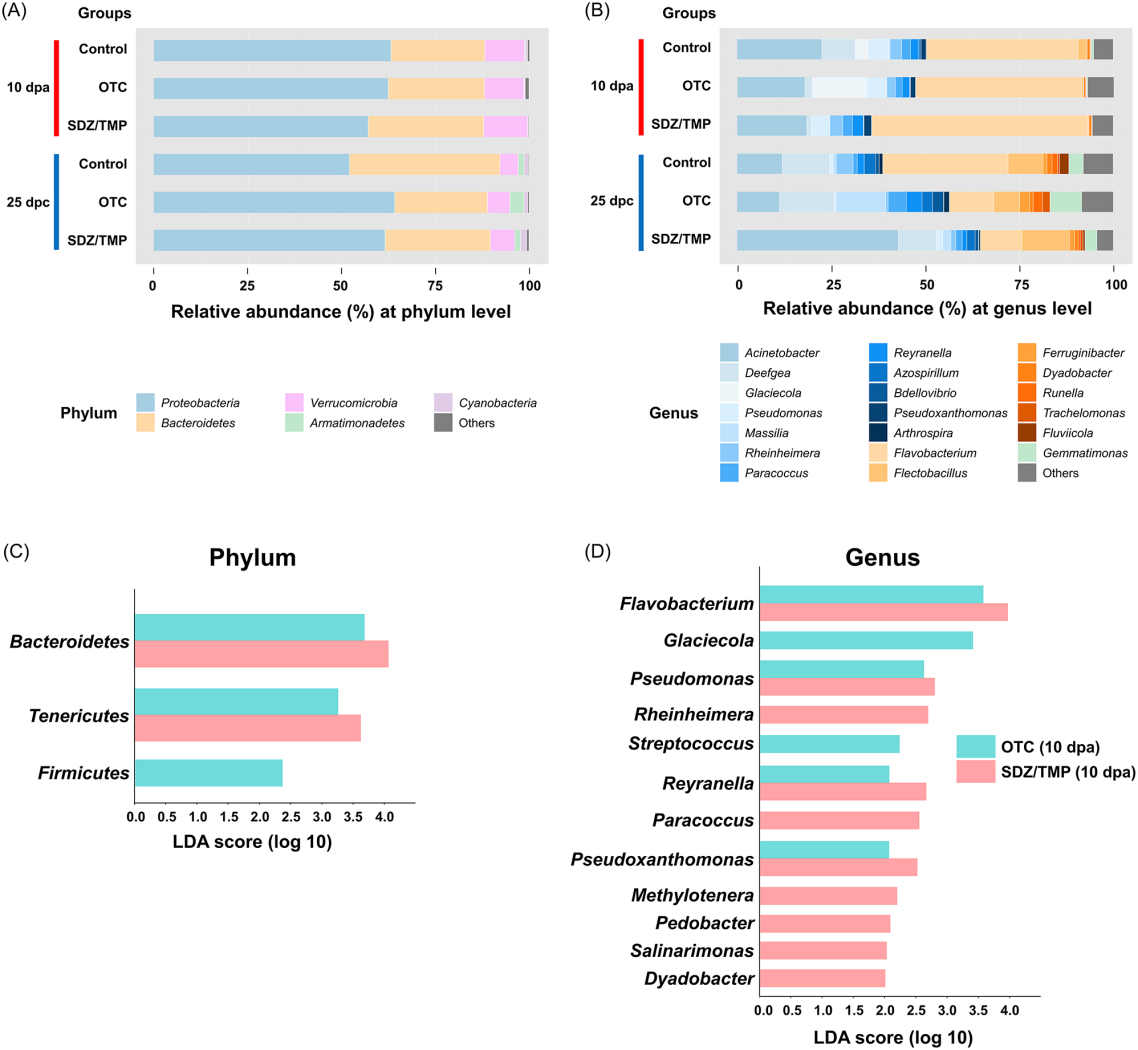
Relative abundance and Linear discriminant analysis effect size (LEfSe) analysis of bacterial taxa. Relative abundance of the 5 most abundant bacterial phyla (**A**) and 20 most abundant bacterial genera (**B**) in control, oxytetracycline (OTC)-treated, and sulfadiazine/trimethoprim (SDZ/TMP)-treated rainbow trout feces. The bar chart displays taxonomic differences in the fecal microbiota at phylum (C) and genus (D) levels between control and oxytetracycline-treated or sulfadiazine/trimethoprim-treated groups of rainbow trout at 10 dpa, showing the log-transformed LDA scores of bacterial taxa identified by LEfSe analysis (LDA threshold score > 2.0 with *p*-value < 0.05)

A total of 327 OTUs were detected and classified into bacterial taxa across all microbiome samples. Of these OTUs, the mean abundance of the top 5 phyla and top 20 genera in the fecal microbiome communities of rainbow trout was presented (Fig. 5). *Proteobacteria*, *Bacteroidetes*, and *Tenericutes* were the dominant phyla in rainbow trout fecal samples (Fig. 5A; Supplementary table S11). In addition, the relative abundance of certain bacterial genera changed over time in response to antibiotic treatment (Fig. 5B; Supplementary table S12). Notably, *Flavobacterium*, which belongs to the *Bacteroidetes* phylum, increased in the fecal microbiome of both antibiotic-treated groups at 10 dpa. At the phylum level, the OTC-treated group showed a significantly higher abundance of *Bacteroidetes* (LDA = 3.66), *Tenericutes* (LDA = 3.26), and *Firmicutes* (LDA = 2.35) compared to the control group (Fig. 5C). Likewise, the SDZ/TMP-treated group had a significantly higher abundance of *Bacteroidetes* (LDA = 4.08), and *Tenericutes* (LDA = 3.64) at 10 dpa. Specifically, at the genus level, *Flavobacterium* (LDA = 3.56 and 3.95 in OTC- and SDZ/TMP-treated groups, respectively), which belongs to *Bacteroidetes*; *Pseudomonas* (LDA = 2.61 and 2.79 in OTC- and SDZ/TMP-treated groups, respectively), *Reyranella* (LDA = 2.07 and 2.65 in OTC- and SDZ/TMP-treated groups, respectively), and *Pseudoxanthomonas* (LDA = 2.06 and 2.51 in OTC- and SDZ/TMP-treated groups, respectively), which belongs to *Proteobacteria*, were significantly enriched in both OTC- and SDZ/TMP-treated groups compared to the control group (Fig. 5D).

### Dynamics in ARGs and *intI1*

**Fig. 6:**
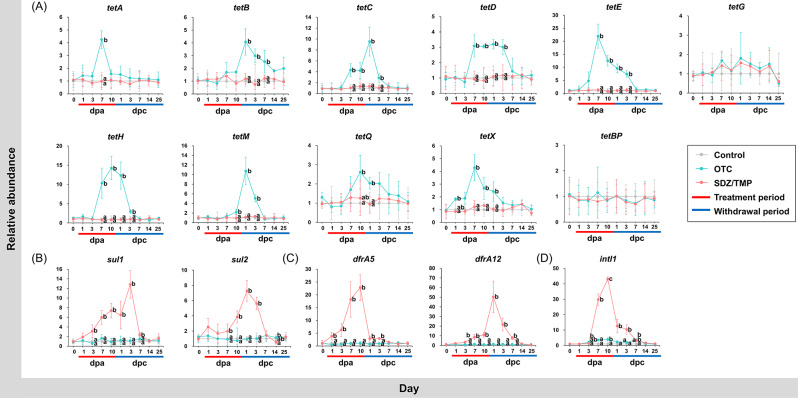
Relative abundance of fifteen antibiotic resistance genes and *intI1* in rainbow trout feces. Relative abundance of tetracycline resistance genes: *tetA*, *tetB*, *tetC*, *tetD*, *tetE*, *tetG*, *tetH*, *tetM*, *tetQ*, *tetX*, *tetBP* (**A**); sulfonamide resistance genes: *sul1*, *sul2* (**B**); trimethoprim resistance genes: *dfrA5*, *dfrA12* (**C**); mobile genetic elements: *intI1* (**D**) in rainbow trout feces before, during, and after oxytetracycline (OTC)- and sulfadiazine/trimethoprim (SDZ/TMP)-treatment was normalized to that of 16S rRNA genes in the respective samples. Error bars indicate the standard deviation of triplicate samples. Groups with different letters were significantly different (*p*-value ≤ 0.05) based on one-way ANOVA test

The relative abundance of ARGs and *intI1* was normalized to the 16S rRNA gene. Antibiotic administration increased the relative abundance of ARGs and *intI1* in the rainbow trout feces (Fig. 6; Supplementary table S13; Supplementary table S14). Tested ARGs and *intI1* were detected in the control group, and the abundance of ARGs and *intI1* in the control group was considered as the background levels for each day. Overall, during the antibiotic treatment period, the relative abundance of tetracycline resistance genes, excluding *tetG* and *tetBP*, increased in the OTC-treated group, while sulfonamide and trimethoprim resistance genes increased in the SDZ/TMP-treated group. The relative abundance of *intI1* also showed distinct patterns between the groups. In the OTC-treated group, *intI1* significantly increased on 3 and 10 dpa, while in the SDZ/TMP-treated group, a significant increase was observed starting at 7 dpa. At 10 dpa, *intI1* reached its maximum abundance in both OTC- and SDZ/TMP-treated groups, with values of 3.89 ± 1.05% and 43.06 ± 1.40%, respectively. Notably, during the withdrawal period, the relative abundance of ARGs and *intI1* in the antibiotic-treated groups gradually decreased. By 14 dpc, the relative abundance of all targeted ARGs and *intI1* had become comparable to that of the control group.

### Correlation of ARGs and *intI1* abundance

The correlation between ARGs and *intI1* was investigated for various antimicrobial classes, with correlation coefficients categorized as weak, moderate, or strong based on their values (0.1 ≤ *r* < 0.4, 0.4 ≤ *r* < 0.7, and 0.7 ≤ *r* < 1.0) according to Akoglu [42] and Schober et al. [43] (Supplementary table S15). Among tetracycline resistance genes, *intI1* showed moderate to strong positive correlations with *tetC*, *tetD*, *tetE*, *tetH*, and *tetX*, with correlation coefficients ranging from 0.43 (*tetE*, *p* < 0.001) to 0.62 (*tetH*, *p* < 0.001). For sulfonamide resistance genes, strong positive correlations were observed between *intI* and both *sul1* (*r* = 0.71) and *sul2* (*r* = 0.73). Likewise, a highly strong positive correlation was observed between trimethoprim resistance genes (*dfrA5*) and *intI1*, with correlation coefficients of 0.92.

### Relationship between ARGs, *intI1*, and microbial community

**Fig. 7:**
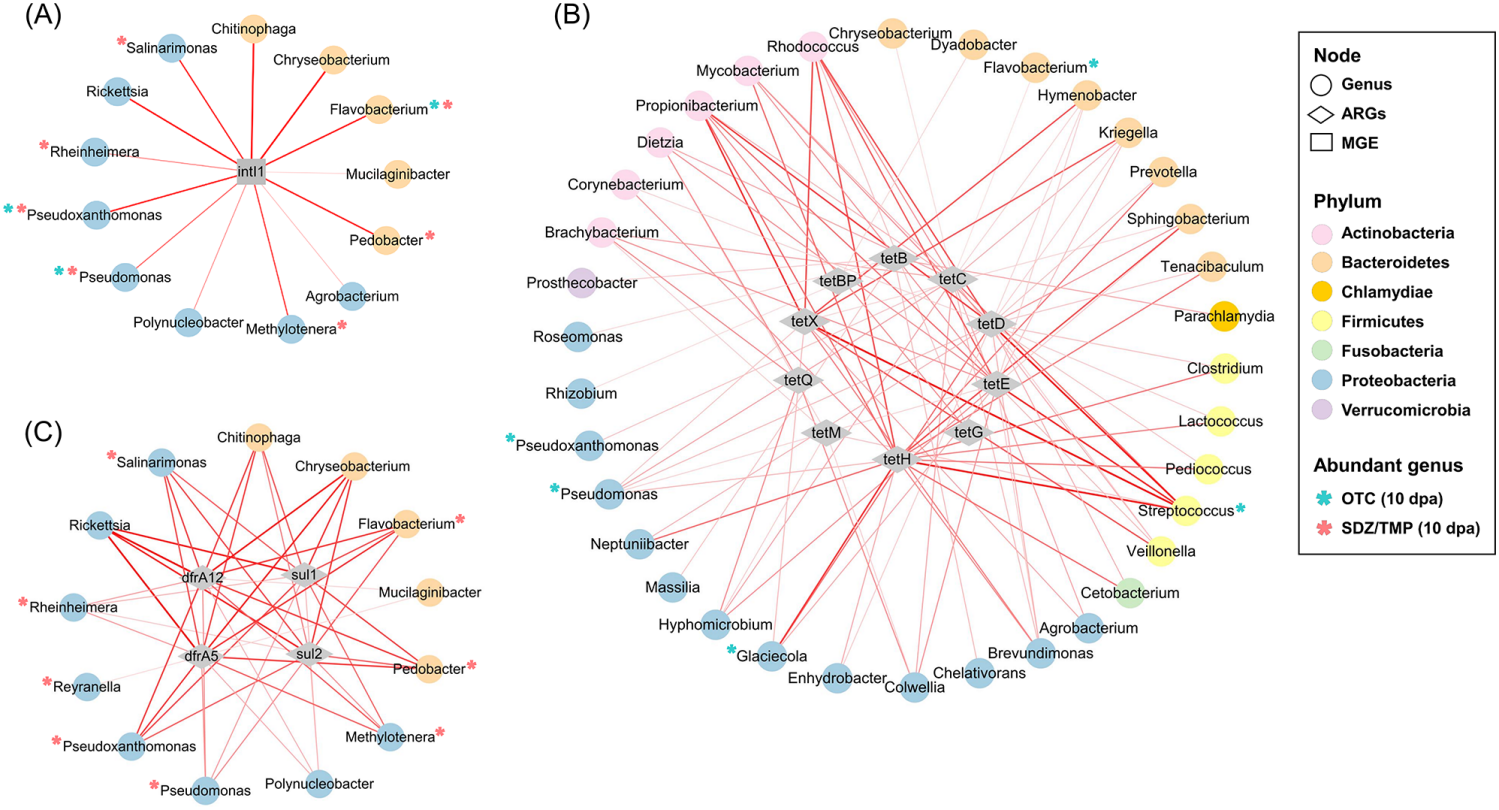
Association network among antibiotic resistance genes (ARGs), mobile genetic element (MGE), and fecal microbiomes. Genera correlated with *intI1* (**A**); with tetracycline resistance genes (**B**); with sulfonamide and trimethoprim resistance genes (**C**) were shown. The edges represented a positive correlation (*p*-value ≤ 0.05) between the connected nodes. The thickness of the edges represented the strength of the correlation, as indicated by Pearson’s correlation coefficients. Asterisks represented differently abundant genera in the fecal microbiota of rainbow trout between control and oxytetracycline (OTC)-treated or sulfadiazine/trimethoprim (SDZ/TMP)-treated groups during the 10-day antibiotics treatment

In the fecal microbiome of rainbow trout, a total of 61 genera belonging to 10 phyla were moderately to strongly associated with ARGs and *intI1*. Particularly, *intI1* had only a positive correlation with 13 genera, predominantly belonging to *Bacteroidetes* and *Proteobacteria* (Fig. 7A). In addition, 35 genera belonging to 6 phyla showed moderate and strong positive associations with tetracycline resistance genes, including 5 genera significantly enriched in the OTC-treated group (Fig. 7B; Supplementary table S16). Among the *Bacteroidetes*, 8 genera showed positive correlations with *tetC*, *tetD*, *tetE*, *tetH*, *tetX*, and *tetBP*. Particularly, *Streptococcus*, a member of the *Firmicutes* phylum, had strong positive correlations with the 5 tetracycline resistance genes (*tetC*, *tetD*, *tetE*, *tetH*, and *tetX*) and a moderate positive correlation with *tetM*. Additionally, *Proteobacteria* showed positive associations with 8 tetracycline resistance genes (*tetB*, *tetC*, *tetD*, *tetE*, *tetH*, *tetM*, *tetQ*, and *tetX*). Within this phylum, *Pseudomonas* had the most diverse correlations with tetracycline resistance genes (*tetC*, *tetD*, *tetE*, *tetH*, *tetX*). On the other hand, 13 genera belonging to *Bacteroidetes* and *Proteobacteria* exhibited moderate to strong positive correlations with sulfonamide and trimethoprim resistance genes, including 8 differentially abundant genera in the SDZ/TMP-treated group (Fig. 7C; Supplementary table S16). Within *Bacteroidetes*, *Pedobacter* and *Flavobacterium* showed strong positive correlations ranging from 0.75 (with *sul2*) to 0.82 (with *dfrA5*) and from 0.76 (with *sul2*) to 0.83 (with *dfrA5*), respectively. Additionally, 8 genera belonging to *Proteobacteria*, except for *Reyranella*, were positively associated with *sul1*, *sul2*, *dfrA5*, and *dfrA12*.

The presence or absence of tetracycline resistance genes in genera enriched in the OTC-treated group was investigated using BLAST search (Supplementary table S17). *Pseudomonas* was shown to have the most diverse tetracycline resistance genes, with *tetC*, *tetD*, *tetE*, and *tetX* showing strong positive correlations. In addition, *Streptococcus* was found to harbor *tetB*, *tetC*, *tetH*, *tetM*, and *tetBP*, and *Flavobacterium* harbored *tetM* and *tetX*. On the other hand, after 10 days of treatment with SDZ/TMP, the presence of sulfonamide resistance genes was confirmed in 5 out of 10 differentially abundant genera, significantly increased in rainbow trout feces. Specifically, *Flavobacterium*, *Pseudomonas*, and *Rheinheimera* possessed and indicated positive correlations with all tested sulfonamide resistance genes. Additionally, *Paracoccus* harbored both *sul1* and *sul2*, and *Pseudoxanthomonas* only had *sul1*. In addition, *Pseudomonas* was found to possess both *dfrA5* and *dfrA12*.

## Discussion

In this study, antibiotics administration led to a significant decrease in the bacterial count cultured from the feces. However, by the 7 dpc, the fecal microbial counts returned to baseline levels, indicating that rainbow trout require at least 7 days to restore their gut microbial numbers after antibiotic intervention. This finding aligns with previous studies. For instance, Sumithra et al. [15] and Kim et al. [11] demonstrated the restoration of total cultivable bacterial counts in the intestine of snubnose pompano and olive flounder *(Paralichthys olivaceus*), respectively, within 10 days following the withdrawal of florfenicol and oxytetracycline treatments. In contrast, our study revealed that the restoration of cultured microbial diversity took a longer duration, and was not achieved until 25 dpc. While the gut or fecal microbial counts of the fish needed approximately 7 to 10 days for recovery, microbial diversity took more than 25 days for recovery.

It is worth noting that culture-based techniques might not fully capture the entire bacterial population present in the feces due to the existence of non-culturable microorganisms [44, 45]. To address this limitation, we employed high-throughput sequencing, which provided a comprehensive understanding of the microbial community dynamics [46]. Since many environmental microbes are difficult to culture, it is unsurprising that the two approaches yield divergent taxonomic profiles [47, 48]. For instance, a study comparing both methods found that only 5.1% of OTUs from the human gut microbiome [49] and 2.4% of OTUs from the soil microbiome [44] were shared between the two techniques. Notably, in this study, some microorganisms detected by culture-dependent methods were absent in culture-independent analyses. This discrepancy may result from primer mismatches or the overestimation of specific taxa in culture-independent datasets [47, 48]. Similar findings have been reported in soil [44] and marine sediment [47] microbiomes, where culturable bacteria were not detected by amplicon sequencing.

The analysis of alpha- and beta-diversity of the fecal microbial communities revealed the antibiotic-induced changes in fecal microbial diversity in this study. Despite the presence of OTC and SDZ/TMP, the initial fecal microbial richness and evenness remained stable, consistent with previous studies on the gut microbiomes of fish treated with oxytetracycline [19, 50]. However, in our study, microbial diversity exhibited a remarkable shift after the cessation of antibiotic treatment. After antibiotic withdrawal, microbial richness significantly increased in both treatment groups, indicating a disruption of colonization resistance and the potential expansion of resident or invasive microbial populations, as described in Limbu et al. [51]. In addition, an interesting observation emerged regarding microbial evenness after antibiotic cessation. After OTC withdrawal, microbial evenness remained unaffected, while the SDZ/TMP-treated group showed a significant decrease. This discrepancy indicates that broad-spectrum antibiotics like OTC affect a wide range of bacteria proportionally, whereas narrow-spectrum antibiotics like SDZ/TMP target specific bacteria, disrupting the microbial population equilibrium. This pattern aligns with previous observations in mice and human fecal microbiomes, where broad-spectrum antibiotics maintained microbial evenness, while narrow-spectrum antibiotics caused significant reductions [52, 53]. Beta-diversity analysis revealed that time influenced rainbow trout fecal microbiome composition, unlike antibiotic treatment factors, as reflected by the *F*-value, which indicates the importance of grouping factors. These findings align with a previous study [20] on rainbow trout, which observed significant changes in microbial diversity over time, regardless of oxytetracycline treatment. Similarly, a study on European seabass (*Dicentrarchus labrax*) showed that gut microbiome compositions were consistent across various antibiotic treatments [54]. These results suggest that the microbiome undergoes natural fluctuations over time, independent of antibiotic classes, highlighting the importance of considering temporal dynamics when interpreting the effects of antibiotic treatment.

In this study, Illumina iSeq sequencing revealed significant changes in microbial composition following OTC and SDZ/TMP treatments. LEfSe analysis identified differences in bacterial taxa abundances between the control and both antibiotic-treated groups. LDA scores quantified each bacterial taxon’s contribution to the differences in microbial composition between groups. At the phylum level, *Bacteroidetes* and *Tenericutes* were significantly enriched in both antibiotic treatment groups compared to the control group (LDA score > 3.2). *Bacteroidetes* have been identified as hosts for various ARGs [55], while *Tenericutes*, which lack cell walls, can accumulate ARGs [56], enabling survival in antibiotic-rich environments [19]. Additionally, the abundance of *Firmicutes* increased in the OTC-treated group, consistent with previous studies on rainbow trout [19] and Nile tilapia [50] treated with OTC. At the genus level, *Flavobacterium* and *Pseudomonas* exhibited increases in both OTC- and SDZ/TMP-treated groups (LDA score > 2.6), while *Streptococcus* showed enrichment only in the OTC-treated group (LDA score = 2.2). These findings align with those of Yu et al. [57], who reported significant enrichment of *Flavobacterium* in the intestine of zebrafish (*Danio rerio*) exposed to oxytetracycline and sulfamethoxazole separately, and Mannan et al. [58], who observed an increase in *Flavobacterium*, *Pseudomonas*, and *Streptococcus* in oxytetracycline-treated Nile tilapia (*Oreochromis niloticus*) intestines. Notably, the enriched genera *Flavobacterium*, *Pseudomonas*, and *Streptococcus* encompass opportunistic pathogenic genera associated with rainbow trout disease [59].

By 25 dpc, the overall microbiome of the OTC- and SDZ/TMP-treated groups remained distinct from the control group, reflecting persistent instability in the gut microbiome even after the antibiotic withdrawal. Similar patterns have been observed in rainbow trout and Nile tilapia fed with OTC for 7 and 8 days, respectively, even after a 2-week withdrawal period [20, 50]. In contrast, studies on catfish and snubnose pompano treated with florfenicol reported a full restoration of the gut microbiome within 7–15 days post-treatment [14, 15]. In this study, the 25-day withdrawal period was designed to allow sufficient time for microbial recovery while providing a timeframe for comparing recovery durations across fish species. However, the persistence of microbial alterations suggests that this period may not have been adequate to fully restore the gut microbiome, highlighting that even short-term antibiotic administration can impose lasting selective pressures. Furthermore, the relevant competent authority in Korea recommends a 30-day withdrawal period for OTC and SDZ/TMP in salmonids [60]. Future investigations should adopt withdrawal durations aligned with this recommendation to better evaluate microbiome resilience and ensure applicability to aquaculture practices.

One of the most intriguing findings of this study is the significant presence of ARGs in fish feces following specific antibiotic treatments. OTC treatment led to an increase in the abundance of *tet* genes, while SDZ/TMP treatment resulted in elevated levels of *sul* and *dfr* genes. Notably, *tet* genes exhibited lower and slower enrichment compared to *sul* and *dfr* genes. A significant increase in *sul* and *dfr* genes occurred between 1 and 7 days (maximum abundance of *dfrA12*, 50.33 ± 16.34), while *tet* genes abundance began to increase on 7–11 days (maximum abundance of *tetE*, 21.96 ± 4.62). Interestingly, this pattern contrasts with the findings of He et al. [61], who observed more rapid and pronounced increases in *tet* genes compared to *sul* genes in rearing water of Crucian carp (*Carassius carassius*) treated with tetracycline and sulfanilamide antibiotics, respectively. Additionally, personal communication with a rainbow trout farmer revealed that OTC is commonly used on the farm, while sulfa drugs are not. This implies that the abundance of *tet* genes might be expected to increase rapidly and abundantly compared to *sul* and *dfr* genes, due to previous exposure to OTC. However, contrary to this expectation, we observed a greater and faster enrichment of *sul* and *dfr* genes. This could be attributed to an increase in bacterial groups harboring these genes, the amplification of sulfonamide and trimethoprim resistance genes [62], and enhanced gene transfer mechanisms.

Horizontal gene transfer (HGT) plays a significant role in the transfer of ARGs in aquatic environments and fish guts [63]. In our study, quantitative analysis of *intI1*, a gene linked to ARGs transfer [64], revealed an increase during antibiotic treatment, indicating HGT occurrences within the bacterial community. Importantly, the abundance of ARGs and *intI1* decreased during the withdrawal period, indicating the fecal microbiota’s ability to recover after cessation of antibiotic exposure. Furthermore, *sul* and *dfr* genes exhibited a strong positive correlation with *intI1*, suggesting their greater susceptibility to transfer compared to *tet* genes in rainbow trout feces. Previous studies have reported the presence of class 1 integron gene cassettes containing sulfonamide and trimethoprim resistance genes [65], and a positive correlation between *intI1* and *sul1* in various ecosystems, such as river and aquaculture farm water [66]. The varying correlations between *intI1* and different antibiotic classes highlight the specific dynamics of HGT mechanisms.

Within association networks, *Bacteroidetes* and *Proteobacteria* exhibited positive correlations with *intI1* and ARGs, consistent with previous findings in antibiotic-contaminated aquaculture systems and hybrid groupers [67, 68]. In the OTC- and SDZ/TMP-treated groups, most of the significantly increased genera (5 out of 6 genera; 8 out of 10 genera, respectively) showed positive correlations with *tet* genes and *sul* and *dfr* genes, respectively. Particularly, opportunistic pathogenic genera such as *Flavobacterium*, *Pseudomonas*, and *Streptococcus* demonstrated strong correlations with ARGs and *intI1* in this study. Additionally, a BLAST search confirmed that they harbored multiple ARGs, consistent with previous findings in the gut of grouper and zebrafish [57, 67]. The release of resistant bacteria carrying ARGs during antibiotic treatment contributes to the spread of antibiotic resistance in the environment [69]. These findings suggest that these pathogenic genera may serve as ARG carriers, facilitating the spread of antibiotic resistance through HGT. Overall, our findings enhance our understanding of the complex interactions between ARGs, MGEs, and the fecal microbiome, highlighting the potential role of specific bacterial taxa as contributors to antibiotic resistance in aquatic environments.

## Conclusion

This study highlights the potential of monitoring antibiotic-induced changes in the microbial community and ARGs abundance in fish feces. Antibiotic treatment disrupts the fecal microbial communities of rainbow trout, leading to a substantial shift in microbial diversity. The concurrent increase in *intI1* and specific bacterial populations correlated with ARGs contributes to elevated ARG abundance in feces. Even after a 25-day withdrawal period, microbial composition and diversity did not stabilize, suggesting prolonged recovery post-antibiotic treatment. Future studies should determine the timeline for fecal microbiome restoration. Overall, fish feces may serve as reservoirs for ARB and ARGs within aquaculture systems, providing a non-invasive approach for tracking antibiotic resistance dynamics. This novel method offers new possibilities for early prediction and monitoring of gut microbiome and resistome changes in aquaculture environments.

## Electronic supplementary material

Below is the link to the electronic supplementary material.

Supplementary Material 1

Supplementary Material 2

## Data Availability

No datasets were generated or analysed during the current study.
