# Supplementary material for: Short-term dynamics of fecal microbiome and antibiotic resistance in juvenile rainbow trout (Oncorhynchus mykiss) following antibiotic treatment and withdrawal
Source: Anim Microbiome. 2024 Dec 20;6:72. doi: 10.1186/s42523-024-00361-0 (PMC11662461; doi:10.1186/s42523-024-00361-0)
Supplement: Supplementary file 1 — Supplementary Material 1 [file 42523_2024_361_MOESM1_ESM.docx]

| 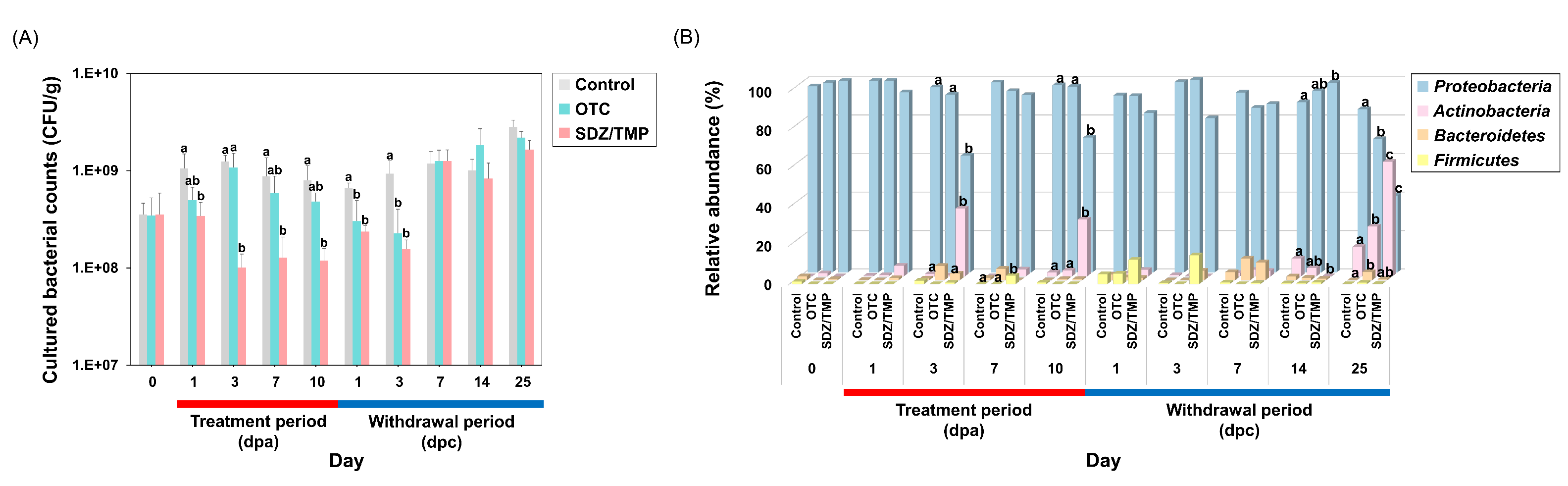 |
| --- |
| **Figure S1**. Changes in counts of cultured bacteria in rainbow trout feces. Feces from control, oxytetracycline (OTC)-, and sulfadiazine/trimethoprim (SDZ/TMP)-treated rainbow trout were analyzed. Abbreviations "dpa" and "dpc" represent “days post antibiotic administration” and “days post antibiotic cessation”, respectively. Statistically significant differences among groups were assessed using a one-way ANOVA followed by Duncan's multiple range test. |
| 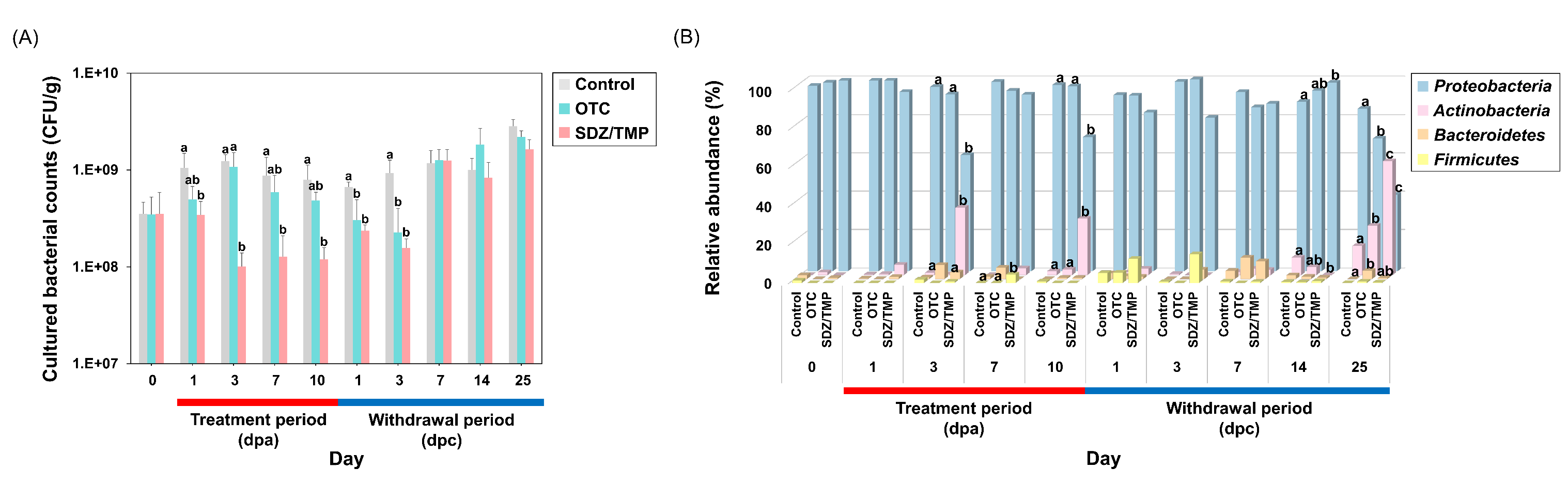 |
| **Figure S2**. Changes in relative abundances of cultured bacteria in rainbow trout feces. Feces from control, oxytetracycline (OTC)-, and sulfadiazine/trimethoprim (SDZ/TMP)-treated rainbow trout were analyzed. Abbreviations "dpa" and "dpc" represent “days post antibiotic administration” and “days post antibiotic cessation”, respectively. Statistically significant differences among groups were assessed using a one-way ANOVA followed by Duncan's multiple range test. |
| 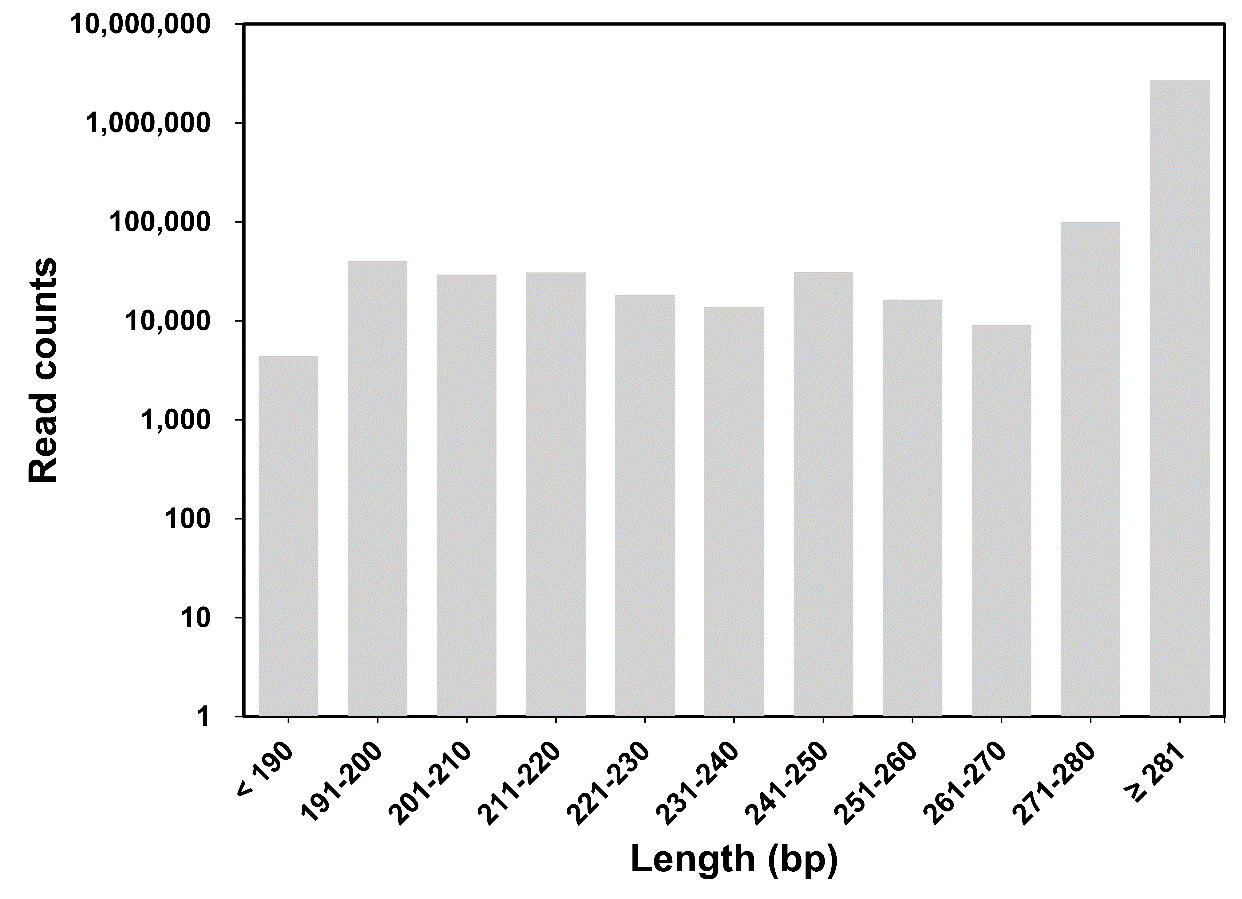 |
| **Figure S3**. Distribution of effective sequence lengths. The x-axis represents the range of sequence length range, and the y-axis indicates the read number. |
| 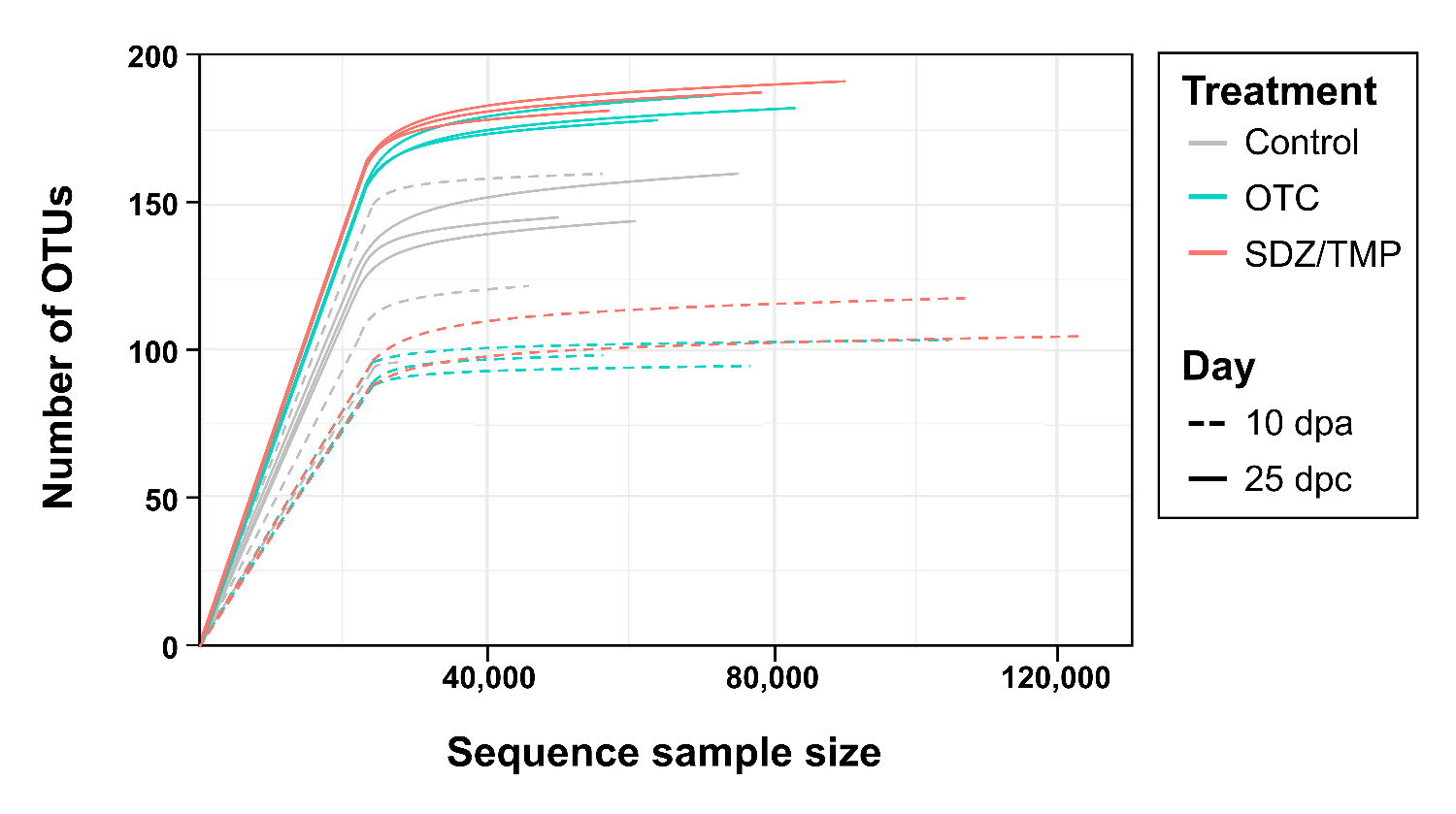 |
| **Figure S4**. Rarefaction curves showing observed OTUs richness in samples. The x-axis represents the sequencing depth in the number of reads obtained from control, oxytetracycline (OTC)-, and sulfadiazine/trimethoprim (SDZ/TMP)-treated rainbow trout feces, and the y-axis is the estimated OTU richness detected in each sample. The rarefaction curves for each group (n=3, except for the SDZ/TMP group on 10 dpa) were displayed by different colors. |
