# Supplementary material for: Short-term dynamics of fecal microbiome and antibiotic resistance in juvenile rainbow trout (Oncorhynchus mykiss) following antibiotic treatment and withdrawal
Source: Anim Microbiome. 2024 Dec 20;6:72. doi: 10.1186/s42523-024-00361-0 (PMC11662461; doi:10.1186/s42523-024-00361-0)
Supplement: Supplementary file 2 — Supplementary Material 2 [file 42523_2024_361_MOESM2_ESM.docx]

| **Table S1**. The list of primer sets used in conventional PCR array.   \| Classification \| No. \| Target gene \| Primer \| Sequence (5'-3') \| Reference \| \| --- \| --- \| --- \| --- \| --- \| --- \| \| Tetracycline resistance genes \| 1 \| *tetA* \| Forward  Reverse \| GCG-CTN-TAT-GCG-TTG-ATG-CA  ACA-GCC-CGT-CAG-GAA-ATT \| Jun et al., 2004 \| \| 2 \| *tetB* \| Forward  Reverse \| GCG-CTN-TAT-GCG-TTG-ATG-CA  TGA-AAG-CAA-ACG-GCC-TAA \| Jun et al., 2004 \| \| 3 \| *tetC* \| Forward  Reverse \| GCG-CTN-TAT-GCG-TTG-ATG-CA  CGT-GCA-AGA-TTC-CGA-ATA \| Jun et al., 2004 \| \| 4 \| *tetD* \| Forward  Reverse \| GCG-CTN-TAT-GCG-TTG-ATG-CA  CCA-GAG-GTT-TAA-GCA-GTG-T \| Jun et al., 2004 \| \| 5 \| *tetE* \| Forward  Reverse \| GCG-CTN-TAT-GCG-TTG-ATG-CA  ATG-TGT-CCT-GGA-TTC-CT \| Jun et al., 2004 \| \| 6 \| *tetG* \| Forward  Reverse \| GCG-CTN-TAT-GCG-TTG-ATG-CA  ATG-CCA-ACA-CCC-CCG-GCG \| Jun et al., 2004 \| \| 7 \| *tetH* \| Forward  Reverse \| ACC-GAT-AGA-AGT-TGT-GTT-CCA-A  CGC-TTG-TTG-CCA-ATA-GGA-CA \| Mao et al., 2015 \| \| 8 \| *tetK* \| Forward  Reverse \| TCG-ATA-GGA-ACA-GCA-GTA  CAG-CAG-ATC-CTA-CTC-CTT \| Srinivasan et al., 2008 \| \| 9 \| *tetL* \| Forward  Reverse \| CCT-GCG-AGT-ACA-AAC-TGG  TCA-AGG-TAA-CCA-GCC-AAC \| Matter et al., 2007 \| \| 10 \| *tetM* \| Forward  Reverse \| GCA-ATT-CTA-CTG-ATT-TCT-GC  CTG-TTT-GAT-TAC-AAT-TTC-CGC \| Tamminen et al., 2011 \| \| 11 \| *tetO* \| Forward  Reverse \| ACG-GAR-AGT-TTA-TTG-TAT-ACC  TGG-CGT-ATC-TAT-AAT-GTT-GAC \| Munir et al., 2011 \| \| 12 \| *tetQ* \| Forward  Reverse \| AGA-ATC-TGC-TGT-TTG-CCA-GTG  CGG-AGT-GTC-AAT-GAT-ATT-GCA \| Aminov et al., 2001 \| \| 13 \| *tetS* \| Forward  Reverse \| CAT-AGA-CAA-GCC-GTT-GAC-C ATG-TTT-TTG-GAA-CGC-CAG-AG \| Srinivasan et al., 2008 \| \| 14 \| *tetV* \| Forward  Reverse \| GCG-GGA-ACG-ACG-ATG-TAT-ATC CCG-CTA-TCT-CAC-GAC-CAT-GAT \| Ouyang et al., 2015 \| \| 15 \| *tetW* \| Forward  Reverse \| GAG-AGC-CTG-CTA-TAT-GCC-AG  GGG-CGT-ATC-CAC-AAT-GTT-AA \| Petrinić, 2020 \| \| 16 \| *tetX* \| Forward  Reverse \| GGA-CCC-GTT-GGA-CTG-ACT-ATG  TAC-ACC-CAT-TGG-TAA-GGC-TAA-GT \| Mao et al., 2015 \| \| 17 \| *tetZ* \| Forward  Reverse \| CCT-TCT-CGA-CCA-GGT-CGG  ACC-CAC-AGC-GTG-TCC-GTC \| Schwaiger et al., 2009 \| \| 18 \| *tetBP* \| Forward  Reverse \| AAA-ACT-TAT-TAT-ATT-ATA-GTG  TGG-AGT-ATC-AAT-AAT-ATT-CAC \| Aminov et al., 2001 \| \| 19 \| *tet34* \| Forward  Reverse \| GCT-TGC-GAT-TAA-TTG-GTT-CC  TTG-GGA-CGC-CAT-GCA-AAA-TT \| Kim et al., 2003 \| \| 20 \| *tet39* \| Forward  Reverse \| CTC-CTT-CTC-TAT-TGT-GGC-TA  CAC-TAA-TAC-CTC-TGG-ACA-TC \| Agersø & Petersen, 2007 \| \| Sulfonamide resistance genes \| 21 \| *sul1* \| Forward  Reverse \| CGG-CGT-GGG-CTA-CCT-GAA-CG  GCC-GAT-CGC-GTG-AAG-TTC-CG \| Kerrn et al., 2002 \| \| 22 \| *sul2* \| Forward  Reverse \| GCG-CTC-AAG-GCA-GAT-GGC-ATT  GCG-TTT-GAT-ACC-GGC-ACC-CGT \| Kerrn et al., 2002 \| \| 23 \| *sul3* \| Forward  Reverse \| TCC-GTT-CAG-CGA-ATT-GGT-GCA-G  TTC-GTT-CAC-GCC-TTA-CAC-CAG-C \| Xiong et al., 2015 \| \| 24 \| *sulA* \| Forward  Reverse \| TCT-TGA-GCA-AGC-ACT-CCA-GCA-G  TCC-AGC-CTT-AGC-AAC-CAC-ATG-G \| Pei et al., 2006 \| \| Trimethoprim resistance genes \| 25 \| *dfrA1* \| Forward  Reverse \| TGG-TAG-CTA-TAT-CGA-AGA-ATG-GAG-T  TAT-GTT-AGA-GGC-GAA-GTC-TTG-GGT-A \| Grape et al., 2007 \| \| 26 \| *dfrA5* \| Forward  Reverse \| AGC-TAC-TCT-TTA-AAG-CCT-TGA-CGT-A  GTG-TTG-CTC-AAA-AAC-AAC-TTC-G \| Grape et al., 2007 \| \| 27 \| *dfrA7* \| Forward  Reverse \| ACA-TTT-GAC-TCT-ATG-GGT-GTT-CTT-C  ACC-TCA-ACG-TGA-ACA-GTA-GAC-AAA-T \| Grape et al., 2007 \| \| 28 \| *dfrA12* \| Forward  Reverse \| GAG-CTG-AGA-TAT-ACA-CTC-TGG-CAC-T  GTA-CGG-AAT-TAC-AGC-TTG-AAT-GGT \| Grape et al., 2007 \| \| 29 \| *dfrA14* \| Forward  Reverse \| CTG-CGA-AAG-CGA-AAA-ACG-GCG  GGA-ATA-CTC-GGG-AAG-AAA-ACA \| Kim et al., 2018 \| \| 30 \| *dfrA17* \| Forward  Reverse \| ACA-TTT-GAC-TCT-ATG-GGT-GTT-CTT-C  TCT-CTG-GCG-GGG-GTC-AAA-TCT-AT \| Grape et al., 2007 \| \| 31 \| *dfrA18* \| Forward  Reverse \| TGG-GTA-AGA-CAC-TCG-TCA-TGG-G  ACT-GCC-GTT-TTC-GAT-AAT-GTG-G \| Okoh & Igbinosa, 2010 \| \| 32 \| *dfrA19* \| Forward  Reverse \| GAG-CTA-ATA-GTC-GCT-GTG  GAT-CAC-AGT-CGA-ACT-CTC \| Matter et al., 2007 \| \| 33 \| *dfrA23* \| Forward  Reverse \| AGA-ATT-CCC-TTC-TCT-TTG-AT  ATG-CCA-ACA-GTT-GAG-ATT-AT \| Amador et al., 2019 \| \| 34 \| *dfrB* \| Forward  Reverse \| GRG-ATC-GCG-TRC-GCA-AGA-ART-C  GCS-GCA-ACA-GGA-TAA-ATC-TG \| Matter et al., 2007 \| |
| --- | --- | --- | --- | --- | --- | --- | --- | --- | --- | --- | --- | --- | --- | --- | --- | --- | --- | --- | --- | --- | --- | --- | --- | --- | --- | --- | --- | --- | --- | --- | --- | --- | --- | --- | --- | --- | --- | --- | --- | --- | --- | --- | --- | --- | --- | --- | --- | --- | --- | --- | --- | --- | --- | --- | --- | --- | --- | --- | --- | --- | --- | --- | --- | --- | --- | --- | --- | --- | --- | --- | --- | --- | --- | --- | --- | --- | --- | --- | --- | --- | --- | --- | --- | --- | --- | --- | --- | --- | --- | --- | --- | --- | --- | --- | --- | --- | --- | --- | --- | --- | --- | --- | --- | --- | --- | --- | --- | --- | --- | --- | --- | --- | --- | --- | --- | --- | --- | --- | --- | --- | --- | --- | --- | --- | --- | --- | --- | --- | --- | --- | --- | --- | --- | --- | --- | --- | --- | --- | --- | --- | --- | --- | --- | --- | --- | --- | --- | --- | --- | --- | --- | --- | --- | --- | --- | --- | --- | --- | --- | --- | --- | --- | --- | --- | --- | --- | --- | --- | --- | --- | --- | --- | --- | --- | --- | --- | --- | --- | --- |
| **Table S2**. The list of primer sets used in qPCR array.   \| Classification \| No. \| Target gene \| Primer \| Sequence (5'-3') \| Reference \| \| --- \| --- \| --- \| --- \| --- \| --- \| \| Tetracycline resistance genes \| 1 \| *tetA* \| Forward  Reverse \| GCT-ACA-TCC-TGC-TTG-CCT-TC  CAT-AGA-TCG-CCG-TGA-AGA-GG \| Ng et al., 2001 \| \| 2 \| *tetB* \| Forward  Reverse \| GGT-TGA-GAC-GCA-ATC-GAA-TT  AGG-CTT-GGA-ATA-CTG-AGT-GTA-A \| Mao et al., 2015 \| \| 3 \| *tetC* \| Forward  Reverse \| CTT-GAG-AGC-CTT-CAA-CCC-AG  ATG-GTC-GTC-ATC-TAC-CTG-CC \| Ng et al., 2001 \| \| 4 \| *tetD* \| Forward  Reverse \| TGA-ACA-GCA-TTC-TCG-CTA-TCA-A  CCA-CTT-GGT-ATA-ACC-CGC-TTT-A \| Mao et al., 2015 \| \| 5 \| *tetE* \| Forward  Reverse \| AAA-CCA-CAT-CCT-CCA-TAC-GC  AAA-TAG-GCC-ACA-ACC-GTC-AG \| Ng et al., 2001 \| \| 6 \| *tetG* \| Forward  Reverse \| GCT-CGG-TGG-TAT-CTC-TGC-TC  AGC-AAC-AGA-ATC-GGG-AAC-AC \| Ng et al., 2001 \| \| 7 \| *tetH* \| Forward  Reverse \| ACC-GAT-AGA-AGT-TGT-GTT-CCA-A  CGC-TTG-TTG-CCA-ATA-GGA-CA \| Mao et al., 2015 \| \| 8 \| *tetM* \| Forward  Reverse \| GCA-ATT-CTA-CTG-ATT-TCT-GC  CTG-TTT-GAT-TAC-AAT-TTC-CGC \| Tamminen et al., 2011 \| \| 9 \| *tetQ* \| Forward  Reverse \| AGA-ATC-TGC-TGT-TTG-CCA-GTG  CGG-AGT-GTC-AAT-GAT-ATT-GCA \| Aminov et al., 2001 \| \| 10 \| *tetX* \| Forward  Reverse \| GGA-CCC-GTT-GGA-CTG-ACT-ATG  TAC-ACC-CAT-TGG-TAA-GGC-TAA-GT \| Mao et al., 2015 \| \| 11 \| *tetBP* \| Forward  Reverse \| AAA-ACT-TAT-TAT-ATT-ATA-GTG  TGG-AGT-ATC-AAT-AAT-ATT-CAC \| Aminov et al., 2001 \| \| Sulfonamide resistance genes \| 12 \| *sul1* \| Forward  Reverse \| CGG-CGT-GGG-CTA-CCT-GAA-CG  GCC-GAT-CGC-GTG-AAG-TTC-CG \| Kerrn et al., 2002 \| \| 13 \| *sul2* \| Forward  Reverse \| GCG-CTC-AAG-GCA-GAT-GGC-ATT  GCG-TTT-GAT-ACC-GGC-ACC-CGT \| Kerrn et al., 2002 \| \| Trimethoprim resistance genes \| 14 \| *dfrA5* \| Forward  Reverse \| AGC-TAC-TCT-TTA-AAG-CCT-TGA-CGT-A  GTG-TTG-CTC-AAA-AAC-AAC-TTC-G \| Grape et al., 2007 \| \| 15 \| *dfrA12* \| Forward  Reverse \| GAG-CTG-AGA-TAT-ACA-CTC-TGG-CAC-T  GTA-CGG-AAT-TAC-AGC-TTG-AAT-GGT \| Grape et al., 2007 \| \| Integrons \| 16 \| *intI1* \| Forward  Reverse \| GCC-TTG-ATG-TTA-CCC-GAG-AG  GAT-CGG-TCG-AAT-GCG-TGT \| Barraud et al., 2010 \| \| 16S rRNA gene \| 17 \| 16S rRNA \| Forward  Reverse \| CCT-ACG-GGA-GGC-AGC-AG ATT-ACC-GCG-GCT-GCT-GG \| Muyzer et al., 1993 \| |

| \| Genus \| NCBI Taxonomy ID \| Number of species \| Number of genomes \| \| --- \| --- \| --- \| --- \| \| *Dyadobacter* \| 120831 \| 26 \| 62 \| \| *Flavobacterium* \| 237 \| 94 \| 1,219 \| \| *Glaciecola* \| 89404 \| 4 \| 48 \| \| *Methylotenera* \| 359407 \| 3 \| 148 \| \| *Paracoccus* \| 265 \| 79 \| 260 \| \| *Pedobacter* \| 84567 \| 74 \| 228 \| \| *Pseudomonas* \| 286 \| 100 \| 30,017 \| \| *Pseudoxanthomonas* \| 83618 \| 22 \| 130 \| \| *Reyranella* \| 445219 \| 3 \| 51 \| \| *Rheinheimera* \| 67575 \| 16 \| 54 \| \| *Salinarimonas* \| 690086 \| 3 \| 4 \| \| *Streptococcus* \| 1301 \| 97 \| 38,434 \|   **Table S3**. List of tested genera and their taxonomy information from National Center for Biotechnology Information (NCBI) platform. Taxonomy information contains their corresponding NCBI Taxonomy IDs, the number of species within each genus, and the number of available genomes. |
| --- | --- | --- | --- | --- | --- | --- | --- | --- | --- | --- | --- | --- | --- | --- | --- | --- | --- | --- | --- | --- | --- | --- | --- | --- | --- | --- | --- | --- | --- | --- | --- | --- | --- | --- | --- | --- | --- | --- | --- | --- | --- | --- | --- | --- | --- | --- | --- | --- | --- | --- | --- | --- |

| **Table S4**. Results of Shapiro-Wilk test for normality and Levene’s test for homogeneity of variances in length and weight of control, oxytetracycline (OTC)-, sulfadiazine/trimethoprim (SDZ/TMP)-treated rainbow trout before, during, and after antibiotic treatment. |
| --- |
| \| Period \| Day \| Test type \| Group \| *p*-value (Length) \| *p*-value (Weight) \| \| --- \| --- \| --- \| --- \| --- \| --- \| \| Pre-treatment period \| 0 \| Normality \| Control \| 0.74 \| 0.05 \| \|  \|  \| OTC \| 0.97 \| 0.93 \| \|  \|  \| SDZ/TMP \| 0.05 \| 0.48 \| \|  \|  \| Homogeneity \| All \| 0.73 \| 0.51 \| \| Treatment period \| 5 \| Normality \| Control \| 0.65 \| 0.66 \| \|  \|  \| OTC \| 0.88 \| 0.85 \| \|  \|  \| SDZ/TMP \| 0.16 \| 0.30 \| \|  \| Homogeneity \| All \| 0.93 \| 0.84 \| \| 10 \| Normality \| Control \| 0.76 \| 0.83 \| \|  \|  \| OTC \| 0.32 \| 0.71 \| \|  \|  \| SDZ/TMP \| 0.25 \| 0.16 \| \|  \|  \| Homogeneity \| All \| 0.85 \| 0.88 \| \| Withdrawal period \| 15 \| Normality \| Control \| 0.21 \| 0.94 \| \|  \|  \| OTC \| 1.00 \| 0.91 \| \|  \|  \| SDZ/TMP \| 0.80 \| 0.05 \| \|  \| Homogeneity \| All \| 0.98 \| 0.81 \| \| 20 \| Normality \| Control \| 0.37 \| 0.85 \| \|  \|  \| OTC \| 0.71 \| 0.90 \| \|  \|  \| SDZ/TMP \| 0.95 \| 0.06 \| \|  \| Homogeneity \| All \| 0.95 \| 0.67 \| \| 25 \| Normality \| Control \| 0.33 \| 0.67 \| \|  \|  \| OTC \| 1.00 \| 0.97 \| \|  \|  \| SDZ/TMP \| 0.97 \| 0.07 \| \|  \| Homogeneity \| All \| 0.77 \| 0.64 \| \| 30 \| Normality \| Control \| 0.45 \| 0.73 \| \|  \|  \| OTC \| 1.00 \| 0.75 \| \|  \|  \| SDZ/TMP \| 0.36 \| 0.19 \| \|  \| Homogeneity \| All \| 0.86 \| 0.63 \| \| 35 \| Normality \| Control \| 0.26 \| 0.41 \| \|  \|  \| OTC \| 0.81 \| 0.96 \| \|  \|  \| SDZ/TMP \| 0.30 \| 0.77 \| \|  \|  \| Homogeneity \| All \| 0.68 \| 0.68 \| |
| **Table S5**. Mean ± standard deviation of length and weight measurements for control, oxytetracycline (OTC)-, sulfadiazine/trimethoprim (SDZ/TMP)-treated rainbow trout before, during, and after antibiotic treatment. |
| \| Period \| Day \| Group \| Length (cm) \| Weight (g) \| \| --- \| --- \| --- \| --- \| --- \| \| Pre-treatment period \| 0 \| Control \| 12.2 ± 0.5 \| 19.68 ± 2.60 \| \|  \| OTC \| 12.1 ± 0.8 \| 20.26 ± 4.30 \| \|  \| SDZ/TMP \| 12.0 ± 0.8 \| 19.88 ± 3.90 \| \| Treatment period \| 5 \| Control \| 12.7 ± 0.6 \| 22.99 ± 4.38 \| \|  \| OTC \| 12.8 ± 0.9 \| 23.93 ± 4.99 \| \|  \| SDZ/TMP \| 12.6 ± 0.8 \| 23.28 ± 3.62 \| \| 10 \| Control \| 13.1 ± 0.8 \| 28.01 ± 5.81 \| \|  \| OTC \| 13.7 ± 0.9 \| 28.49 ± 4.87 \| \|  \| SDZ/TMP \| 13.3 ± 0.7 \| 27.78 ± 3.73 \| \| Withdrawal period \| 15 \| Control \| 13.8 ± 0.9 \| 32.29 ± 7.18 \| \|  \| OTC \| 14.2 ± 0.9 \| 33.36 ± 4.85 \| \|  \| SDZ/TMP \| 14.1 ± 0.9 \| 32.12 ± 4.57 \| \| 20 \| Control \| 14.5 ± 0.9 \| 39.30 ± 9.00 \| \|  \| OTC \| 14.9 ± 0.9 \| 40.25 ± 5.07 \| \|  \| SDZ/TMP \| 14.8 ± 0.8 \| 39.31 ± 4.96 \| \| 25 \| Control \| 15.4 ± 1.1 \| 46.34 ± 10.14 \| \|  \| OTC \| 15.6 ± 0.8 \| 45.35 ± 5.43 \| \|  \| SDZ/TMP \| 15.4 ± 0.8 \| 44.91 ± 5.56 \| \| 30 \| Control \| 15.7 ± 1.0 \| 50.66 ± 11.01 \| \|  \| OTC \| 16.0 ± 0.8 \| 50.88 ± 6.19 \| \|  \| SDZ/TMP \| 16.0 ± 1.0 \| 50.74 ± 6.09 \| \| 35 \| Control \| 16.9 ± 1.3 \| 57.18 ± 12.32 \| \|  \| OTC \| 16.9 ± 0.9 \| 56.66 ± 6.76 \| \|  \| SDZ/TMP \| 17.3 ± 0.8 \| 57.16 ± 6.56 \| |

| **Table S6**. Results of Shapiro-Wilk test for normality and Levene’s test for homogeneity of variances in viable bacterial counts of control, oxytetracycline (OTC)-, sulfadiazine/trimethoprim (SDZ/TMP)-treated rainbow trout before, during, and after antibiotic treatment.   \| Period \| Day \| Test type \| Group \| *p*-value \| \| --- \| --- \| --- \| --- \| --- \| \| Pre-treatment period \| 0 \| Normality \| Control \| 0.80 \| \|  \|  \| OTC \| 0.21 \| \|  \|  \| SDZ/TMP \| 0.98 \| \|  \|  \| Homogeneity \| All \| 0.73 \| \| Treatment period \| 1 \| Normality \| Control \| 0.80 \| \|  \|  \| OTC \| 0.21 \| \|  \|  \| SDZ/TMP \| 0.44 \| \|  \| Homogeneity \| All \| 0.40 \| \| 3 \| Normality \| Control \| 0.40 \| \|  \|  \| OTC \| 0.11 \| \|  \|  \| SDZ/TMP \| 0.84 \| \|  \| Homogeneity \| All \| 0.55 \| \| 7 \| Normality \| Control \| 0.77 \| \|  \|  \| OTC \| 0.30 \| \|  \|  \| SDZ/TMP \| 0.31 \| \|  \| Homogeneity \| All \| 0.39 \| \| 10 \| Normality \| Control \| 0.47 \| \|  \|  \| OTC \| 0.64 \| \|  \|  \| SDZ/TMP \| 0.47 \| \|  \|  \| Homogeneity \| All \| 0.33 \| \| Withdrawal period \| 11 \| Normality \| Control \| 0.36 \| \|  \|  \| OTC \| 0.25 \| \|  \|  \| SDZ/TMP \| 0.84 \| \|  \| Homogeneity \| All \| 0.54 \| \| 13 \| Normality \| Control \| 0.62 \| \|  \|  \| OTC \| 0.05 \| \|  \|  \| SDZ/TMP \| 0.25 \| \|  \| Homogeneity \| All \| 0.40 \| \| 17 \| Normality \| Control \| 0.96 \| \|  \|  \| OTC \| 0.50 \| \|  \|  \| SDZ/TMP \| 0.44 \| \|  \| Homogeneity \| All \| 0.99 \| \| 24 \| Normality \| Control \| 0.05 \| \|  \|  \| OTC \| 0.67 \| \|  \|  \| SDZ/TMP \| 0.36 \| \|  \| Homogeneity \| All \| 0.46 \| \| 35 \| Normality \| Control \| 0.24 \| \|  \|  \| OTC \| 0.70 \| \|  \|  \| SDZ/TMP \| 0.95 \| \|  \|  \| Homogeneity \| All \| 0.83 \| |
| --- | --- | --- | --- | --- | --- | --- | --- | --- | --- | --- | --- | --- | --- | --- | --- | --- | --- | --- | --- | --- | --- | --- | --- | --- | --- | --- | --- | --- | --- | --- | --- | --- | --- | --- | --- | --- | --- | --- | --- | --- | --- | --- | --- | --- | --- | --- | --- | --- | --- | --- | --- | --- | --- | --- | --- | --- | --- | --- | --- | --- | --- | --- | --- | --- | --- | --- | --- | --- | --- | --- | --- | --- | --- | --- | --- | --- | --- | --- | --- | --- | --- | --- | --- | --- | --- | --- | --- | --- | --- | --- | --- | --- | --- | --- | --- | --- | --- | --- | --- | --- | --- | --- | --- | --- | --- | --- | --- | --- | --- | --- | --- | --- | --- | --- | --- | --- | --- | --- | --- | --- | --- | --- | --- | --- | --- | --- | --- | --- | --- | --- | --- | --- | --- | --- | --- | --- | --- | --- | --- | --- | --- | --- | --- | --- | --- | --- | --- | --- | --- | --- | --- | --- | --- | --- | --- | --- | --- | --- | --- | --- | --- | --- | --- | --- | --- | --- | --- | --- | --- | --- | --- |

| **Table S7**. Bacterial species isolated from rainbow trout feces during the experiment period. The cultured bacteria sequences were assigned using Greengenes database, and then they were identified by comparing them with the closest type strains obtained from BLAST analysis. The corresponding accession numbers for the 16S rRNA gene sequences of the bacterial isolates submitted to the NCBI GenBank are listed. |
| --- |
| \| Most closely related species \| Accession number \| Isolation of bacterial cultures \| \| \| \| \| \| \| \| \| \| --- \| --- \| --- \| --- \| --- \| --- \| --- \| --- \| --- \| --- \| --- \| \| Pre-treatment period  (Day 0) \| \| \| Treatment period  (1 to 10 dpa) \| \| \| Withdrawal period  (1 to 25 dpc) \| \| \| \| Control \| OTC \| SDZ/TMP \| Control \| OTC \| SDZ/TMP \| Control \| OTC \| SDZ/TMP \| \| *Actinobacteria* \|  \|  \|  \|  \|  \|  \|  \|  \|  \|  \| \| *Agromyces mediolanus* \| PP967734 \| - \| - \| - \| + \| - \| + \| + \| + \| + \| \| *Leucobacter chromiireducens* \| PP967747-PP967748 \| - \| - \| - \| + \| + \| + \| + \| + \| + \| \| *Microbacterium oxydans* \| PP967751-PP967752 \| - \| + \| - \| + \| + \| + \| + \| + \| + \| \| *Rhodococcus fascians* \| PP967763 \| - \| - \| - \| - \| - \| - \| + \| + \| + \| \| *Rhodococcus qingshengii* \| PP967764-PP967765 \| - \| - \| - \| - \| - \| - \| + \| + \| + \| \| *Bacteroidetes* \|  \|  \|  \|  \|  \|  \|  \|  \|  \|  \| \| *Chryseobacterium formosense* \| PP967739 \| + \| - \| - \| + \| + \| + \| + \| + \| + \| \| *Flavobacterium johnsoniae* \| PP967743 \| - \| - \| - \| - \| - \| + \| - \| - \| + \| \| *Flavobacterium lindanitolerans* \| PP967744 \| + \| - \| + \| + \| + \| + \| + \| + \| - \| \| *Pedobacter africanus* \| PP967753 \| - \| - \| - \| - \| - \| - \| + \| + \| - \| \| *Firmicutes* \|  \|  \|  \|  \|  \|  \|  \|  \|  \|  \| \| *Bacillus safensis* \| PP967735-PP967736 \| + \| - \| - \| + \| - \| + \| + \| + \| + \| \| *Fictibacillus phosphorivorans* \| PP967742 \| - \| - \| - \| - \| - \| + \| - \| - \| + \| \| *Mesobacillus thioparans* \| PP967750 \| - \| - \| - \| - \| - \| - \| + \| + \| + \| \|  \|  \|  \|  \|  \|  \|  \|  \|  \|  \|  \| \|  \|  \|  \|  \|  \|  \|  \|  \|  \|  \|  \| \|  \|  \|  \|  \|  \|  \|  \|  \|  \|  \|  \| \| *Proteobacteria* \|  \|  \|  \|  \|  \|  \|  \|  \|  \|  \| \| *Acidovorax soli* \| PP967724 \| + \| + \| - \| + \| + \| + \| + \| + \| + \| \| *Acinetobacter johnsonii* \| PP967725-PP967730 \| + \| + \| + \| + \| + \| + \| + \| + \| + \| \| *Aeromonas media* \| PP967731 \| - \| - \| + \| + \| + \| + \| + \| + \| + \| \| *Agrobacterium pusense* \| PP967732 \| - \| - \| - \| + \| - \| - \| + \| + \| + \| \| *Agrobacterium radiobacter* \| PP967733 \| + \| + \| + \| + \| + \| + \| + \| + \| + \| \| *Bosea robiniae* \| PP967737 \| - \| - \| - \| - \| - \| + \| + \| + \| + \| \| *Brevundimonas nasdae* \| PP967738 \| - \| - \| - \| - \| + \| + \| - \| - \| + \| \| *Comamonas guangdongensis* \| PP967740 \| + \| - \| + \| + \| - \| + \| + \| + \| + \| \| *Deefgea chitinilytica* \| PP967741 \| + \| + \| + \| + \| + \| + \| + \| + \| + \| \| *Hydrogenophaga palleronii* \| PP967745 \| - \| - \| - \| - \| - \| - \| + \| + \| + \| \| *Ignatzschineria rhizosphaerae* \| PP967746 \| - \| - \| - \| + \| + \| - \| - \| - \| - \| \| *Lysobacter gummosus* \| PP967749 \| - \| - \| - \| - \| - \| - \| + \| + \| + \| \| *Pseudomonas anguilliseptica* \| PP967754 \| - \| - \| - \| + \| - \| + \| - \| + \| + \| \| *Pseudomonas koreensis* \| PP967755-PP967757 \| + \| - \| - \| + \| + \| + \| + \| + \| - \| \| *Pseudomonas peli* \| PP967758 \| - \| - \| - \| + \| + \| + \| + \| + \| + \| \| *Rheinheimera arenilitoris* \| PP967759 \| + \| + \| + \| - \| + \| + \| - \| - \| - \| \| *Rheinheimera chironomi* \| PP967760 \|  \|  \|  \| + \|  \| + \| - \| - \| - \| \| *Rheinheimera soli* \| PP967761-PP967762 \| + \| + \| + \| + \| + \| + \| + \| + \| + \| \| *Shinella zoogloeoides* \| PP967766-PP967769 \| + \| + \| + \| - \| + \| + \| + \| + \| + \| \| *Stenotrophomonas rhizophila* \| PP967770 \| - \| - \| - \| - \| - \| + \| + \| + \| + \| \| *Variovorax paradoxus* \| PP967771 \| + \| + \| + \| + \| + \| + \| + \| + \| + \| |

**Table S8**. Results (*p*-value) of Shapiro-Wilk test for normality and Levene’s test for homogeneity of variances in the abundance of cultured bacterial phyla in rainbow trout feces from the control, oxytetracycline (OTC)-, and sulfadiazine/trimethoprim (SDZ/TMP)-treated groups before, during, and after antibiotic treatment. ‘NA’ indicates unavailable data where the phylum is absent in one or more samples.

| Period | Day | Test type | Group | Phylum |  |  |  |
| --- | --- | --- | --- | --- | --- | --- | --- |
|  |  |  |  | *Proteobacteria* | *Actinobacteria* | *Bacteroidetes* | *Firmicutes* |
| Pre-treatment period | 0 | Normality | Control | 0.49 | NA | 0.95 | 0.05 |
|  |  |  | OTC | 0.05 | 0.05 | NA | NA |
|  |  |  | SDZ/TMP | 0.05 | NA | 0.05 | NA |
|  |  | Homogeneity | All | 0.59 | 0.42 | 0.25 | 0.42 |
| Treatment period | 1 | Normality | Control | 0.57 | 0.05 | 0.05 | NA |
|  |  |  | OTC | 0.05 | 0.05 | NA | NA |
|  |  |  | SDZ/TMP | 0.37 | 0.05 | 0.05 | NA |
|  |  | Homogeneity | All | 0.28 | 0.48 | 0.49 | NA |
|  | 3 | Normality | Control | 0.21 | 0.09 | 0.05 | 0.27 |
|  |  |  | OTC | 0.51 | 0.05 | 0.57 | NA |
|  |  |  | SDZ/TMP | 0.61 | 0.43 | 0.05 | 0.05 |
|  |  | Homogeneity | All | 0.66 | 0.26 | 0.69 | 0.46 |
|  | 7 | Normality | Control | 0.77 | NA | 0.77 | NA |
|  |  |  | OTC | 0.35 | NA | 0.35 | NA |
|  |  |  | SDZ/TMP | 0.34 | 0.05 | NA | 0.96 |
|  |  | Homogeneity | All | 0.53 | 0.42 | 0.31 | 0.08 |
|  | 10 | Normality | Control | 1.00 | 0.27 | NA | 0.05 |
|  |  |  | OTC | 0.74 | 0.26 | 0.05 | NA |
|  |  |  | SDZ/TMP | 0.15 | 0.05 | 0.90 | NA |
|  |  | Homogeneity | All | 0.41 | 0.54 | 0.53 | 0.42 |
| Withdrawal period | 11 | Normality | Control | 0.25 | NA | 0.12 | 0.38 |
|  |  |  | OTC | 0.23 | 0.05 | 0.05 | 0.74 |
|  |  |  | SDZ/TMP | 0.67 | 0.05 | 0.05 | 0.09 |
|  |  | Homogeneity | All | 0.90 | 0.57 | 0.99 | 0.78 |
|  | 13 | Normality | Control | 0.05 | 0.05 | NA | 0.05 |
|  |  |  | OTC | NA | NA | NA | NA |
|  |  |  | SDZ/TMP | 0.05 | NA | 0.13 | 0.05 |
|  |  | Homogeneity | All | 0.44 | 0.42 | 0.35 | 0.44 |
|  | 17 | Normality | Control | 0.44 | 0.05 | 0.77 | 0.68 |
|  |  |  | OTC | 0.05 | 0.27 | 0.18 | NA |
|  |  |  | SDZ/TMP | 0.20 | 0.35 | 0.39 | 0.05 |
|  |  | Homogeneity | All | 0.94 | 0.94 | 0.69 | 0.52 |
|  | 24 | Normality | Control | 0.23 | 0.66 | 0.77 | 0.05 |
|  |  |  | OTC | 0.94 | 0.59 | 0.50 | 0.05 |
|  |  |  | SDZ/TMP | 1.00 | NA | 0.84 | 0.88 |
|  |  | Homogeneity | All | 0.43 | 0.30 | 0.55 | 0.91 |
|  | 35 | Normality | Control | 0.64 | 0.64 | NA | NA |
|  |  |  | OTC | 0.67 | 0.43 | 0.49 | 0.05 |
|  |  |  | SDZ/TMP | 0.44 | 0.34 | 0.90 | 0.05 |
|  |  | Homogeneity | All | 0.27 | 0.54 | 0.21 | 0.50 |

**Table S9**. Mean ± standard deviation of the abundance (%) of cultured bacterial phyla in the feces of rainbow trout from the control, oxytetracycline (OTC)-, and sulfadiazine/trimethoprim (SDZ/TMP)-treated groups before, during, and after antibiotic treatment.

| Period | Day | Group | Phylum |  |  |  |
| --- | --- | --- | --- | --- | --- | --- |
|  |  |  | *Proteobacteria* | *Actinobacteria* | *Bacteroidetes* | *Firmicutes* |
| Pre-treatment period | 0 | Control | 96.62 ± 4.14 | 0.00 | 2.04 ± 2.00 | 1.33 ± 2.31 |
|  |  | OTC | 98.45 ± 2.69 | 1.55 ± 2.69 | 0.00 | 0.00 |
|  |  | SDZ/TMP | 99.44 ± 0.98 | 0.00 | 0.56 ± 0.98 | 0.00 |
| Treatment period | 1 | Control | 99.45 ± 0.51 | 0.33 ± 0.58 | 0.22 ± 0.38 | 0.00 |
|  |  | OTC | 99.43 ± 1.00 | 0.57 ± 1.00 | 0.00 | 0.00 |
|  |  | SDZ/TMP | 93.45 ± 8.63 | 5.44 ± 9.43 | 1.11 ± 1.92 | 0.00 |
|  | 3 | Control | 96.14 ± 4.63 | 1.18 ± 1.03 | 0.92 ± 1.59 | 1.76 ± 2.47 |
|  |  | OTC | 92.20 ± 6.45 | 0.42 ± 0.72 | 7.39 ± 5.76 | 0.00 |
|  |  | SDZ/TMP | 60.59 ± 10.64 | 35.19 ± 8.57 | 3.49 ± 6.05 | 0.73 ± 1.26 |
|  | 7 | Control | 98.72 ± 1.40 | 0.00 | 1.28 ± 1.40 | 0.00 |
|  |  | OTC | 94.11 ± 5.19 | 0.00 | 5.89 ± 5.19 | 0.00 |
|  |  | SDZ/TMP | 92.04 ± 8.74 | 3.51 ± 6.08 | 0.00 | 4.45 ± 2.96 |
|  | 10 | Control | 97.17 ± 0.50 | 2.05 ± 1.80 | 0.00 | 0.78 ± 1.34 |
|  |  | OTC | 96.45 ± 1.49 | 2.99 ± 1.87 | 0.56 ± 0.96 | 0.00 |
|  |  | SDZ/TMP | 69.97 ± 10.16 | 29.44 ± 10.10 | 0.58 ± 0.57 | 0.00 |
| Withdrawal period | 11 | Control | 91.86 ± 9.12 | 0.00 | 2.94 ± 2.41 | 5.20 ± 6.77 |
|  |  | OTC | 91.53 ± 7.39 | 1.52 ± 2.62 | 1.52 ± 2.62 | 5.44 ± 5.93 |
|  |  | SDZ/TMP | 82.81 ± 11.04 | 3.33 ± 5.77 | 1.23 ± 2.14 | 12.62 ± 14.34 |
|  | 13 | Control | 98.83 ± 2.03 | 0.58 ± 1.01 | 0.00 | 0.58 ± 1.01 |
|  |  | OTC | 100.0 ± 0.00 | 0.00 | 0.00 | 0.00 |
|  |  | SDZ/TMP | 80.05 ± 21.70 | 0.00 | 4.95 ± 4.29 | 15.00 ± 25.98 |
|  | 17 | Control | 93.33 ± 6.78 | 1.68 ± 2.91 | 4.22 ± 3.15 | 0.77 ± 0.85 |
|  |  | OTC | 85.43 ± 5.53 | 3.36 ± 1.78 | 11.21 ± 3.79 | 0.00 |
|  |  | SDZ/TMP | 87.38 ± 8.06 | 2.77 ± 1.91 | 9.27 ± 7.46 | 0.59 ± 1.02 |
|  | 24 | Control | 88.33 ± 5.82 | 9.20 ± 3.86 | 1.99 ± 2.16 | 0.48 ± 0.82 |
|  |  | OTC | 94.17 ± 4.64 | 4.21 ± 4.92 | 1.26 ± 1.56 | 0.37 ± 0.64 |
|  |  | SDZ/TMP | 98.38 ± 0.19 | 0.00 | 0.75 ± 0.73 | 0.87 ± 0.91 |
|  | 35 | Control | 84.72 ± 1.83 | 15.28 ± 1.83 | 0.00 | 0.00 |
|  |  | OTC | 69.22 ± 10.37 | 25.81 ± 7.14 | 4.26 ± 3.51 | 0.71 ± 1.23 |
|  |  | SDZ/TMP | 39.86 ± 3.04 | 59.42 ± 3.64 | 0.54 ± 0.55 | 0.17 ± 0.30 |

| **Table S10**. Good’s coverage of rainbow trout fecal samples. Each value represents the mean ± standard deviation of good’s coverage. |
| --- |
| \| Day \| Group \| Good’s coverage (%) \| \| --- \| --- \| --- \| \| 10 dpa \| Control \| 99.99 ± 0.002 \| \| OTC \| 100.00 ± 0.001 \| \| SDZ/TMP \| 99.99 ± 0.001 \| \| 25 dpc \| Control \| 99.99 ± 0.002 \| \| OTC \| 99.99 ± 0.002 \| \| SDZ/TMP \| 99.99 ± 0.001 \| |

**Table S11**. Mean ± standard deviation of the abundance (%) of 5 most abundant bacterial phyla in the feces of rainbow trout from the control, oxytetracycline (OTC)-, and sulfadiazine/trimethoprim (SDZ/TMP)-treated groups.

| Phylum | 10 dpa | | | 25 dpc | | |
| --- | --- | --- | --- | --- | --- | --- |
|  | Control | OTC | SDZ/TMP | Control | OTC | SDZ/TMP |
| *Bacteroidetes* | 24.98 ± 2.87 | 25.62 ± 1.57 | 30.68 ± 0.59 | 40.06 ± 1.02 | 24.64 ± 0.97 | 28.01 ± 0.59 |
| *Gemmatimonadetes* | 0.26 ± 0.33 | 0.09 ± 0.05 | 0.02 ± 0.01 | 1.58 ± 0.22 | 3.75 ± 0.19 | 1.60 ± 0.28 |
| *Proteobacteria* | 63.11 ± 2.97 | 62.52 ± 2.27 | 57.21 ± 0.10 | 52.16 ± 0.75 | 64.23 ± 0.72 | 61.69 ± 1.17 |
| *Tenericutes* | 10.56 ± 1.13 | 10.50 ± 0.63 | 11.72 ± 0.48 | 4.85 ± 0.83 | 5.99 ± 0.74 | 6.48 ± 1.57 |
| *Verrucomicrobia* | 0.51 ± 0.38 | 0.20 ± 0.05 | 0.17 ± 0.00 | 1.12 ± 0.05 | 0.99 ± 0.12 | 1.58 ± 0.08 |

**Table S12**. Mean ± standard deviation of the abundance (%) of 20 most abundant bacterial genera in the feces of rainbow trout from the control, oxytetracycline (OTC)-, and sulfadiazine/trimethoprim (SDZ/TMP)-treated groups.

| Genus | 10 dpa | | | 25 dpc | | |
| --- | --- | --- | --- | --- | --- | --- |
|  | Control | OTC | SDZ/TMP | Control | OTC | SDZ/TMP |
| *Bacteroidetes* |  |  |  |  |  |  |
| *Dyadobacter* | 0.63 ± 0.09 | 0.56 ± 0.22 | 0.72 ± 0.08 | 1.38 ± 0.18 | 0.97 ± 0.31 | 0.93 ± 0.08 |
| *Ferruginibacter* | 0.25 ± 0.28 | 0.08 ± 0.10 | 0.02 ± 0.01 | 0.98 ± 0.19 | 2.61 ± 0.20 | 1.38 ± 0.09 |
| *Flavobacterium* | 40.18 ± 14.48 | 43.97 ± 8.19 | 57.35 ± 0.11 | 33.29 ± 2.45 | 11.76 ± 0.59 | 11.00 ± 0.61 |
| *Flectobacillus* | 2.20 ± 2.52 | 0.47 ± 0.24 | 0.27 ± 0.01 | 9.29 ± 0.89 | 6.90 ± 0.69 | 12.65 ± 1.66 |
| *Fluviicola* | 0.12 ± 0.09 | 0.13 ± 0.04 | 0.10 ± 0.01 | 2.44 ± 0.17 | 0.04 ± 0.01 | 0.53 ± 0.03 |
| *Runella* | 0.22 ± 0.14 | 0.02 ± 0.03 | 0.02 ± 0.00 | 1.48 ± 0.20 | 2.47 ± 0.13 | 0.55 ± 0.02 |
| *Trachelomonas* | 0.13 ± 0.17 | 0.08 ± 0.08 | 0.01 ± 0.01 | 0.54 ± 0.13 | 2.02 ± 0.37 | 0.74 ± 0.13 |
| *Gemmatimonadetes* |  |  |  |  |  |  |
| *Gemmatimonas* | 0.62 ± 0.75 | 0.23 ± 0.13 | 0.04 ± 0.03 | 3.87 ± 0.62 | 8.34 ± 0.38 | 3.14 ± 0.52 |
| *Proteobacteria* |  |  |  |  |  |  |
| *Acinetobacter* | 22.67 ± 8.19 | 17.94 ± 0.95 | 18.64 ± 0.26 | 12.15 ± 0.89 | 11.37 ± 1.42 | 42.92 ± 2.93 |
| *Arthrospira* | 0.15 ± 0.10 | 0.14 ± 0.04 | 0.12 ± 0.00 | 0.94 ± 0.24 | 1.44 ± 0.07 | 0.37 ± 0.06 |
| *Azospirillum* | 0.42 ± 0.59 | 0.05 ± 0.06 | 0.01 ± 0.01 | 3.04 ± 0.47 | 2.79 ± 0.51 | 2.20 ± 0.62 |
| *Bdellovibrio* | 0.30 ± 0.32 | 0.16 ± 0.11 | 0.11 ± 0.01 | 0.98 ± 0.12 | 2.91 ± 0.35 | 0.90 ± 0.08 |
| *Deefgea* | 8.86 ± 5.52 | 1.97 ± 0.55 | 1.17 ± 0.10 | 12.63 ± 2.70 | 14.41 ± 0.57 | 10.15 ± 0.93 |
| *Glaciecola* | 3.37 ± 2.71 | 14.46 ± 11.18 | 0.00 | 0.01 ± 0.01 | 0.00 | 0.00 |
| *Massilia* | 0.33 ± 0.57 | 0.00 | 0.00 | 0.84 ± 0.17 | 13.11 ± 0.70 | 2.18 ± 0.05 |
| *Paracoccus* | 2.30 ± 0.20 | 1.83 ± 0.36 | 2.52 ± 0.05 | 1.12 ± 0.10 | 4.85 ± 0.82 | 1.72 ± 0.08 |
| *Pseudomonas* | 5.51 ± 1.59 | 5.32 ± 0.82 | 4.95 ± 0.06 | 0.84 ± 0.08 | 0.64 ± 0.11 | 1.62 ± 0.18 |
| *Pseudoxanthomonas* | 1.34 ± 0.75 | 1.36 ± 0.27 | 1.97 ± 0.03 | 0.00 | 0.14 ± 0.03 | 0.12 ± 0.03 |
| *Reyranella* | 2.12 ± 0.58 | 1.97 ± 0.57 | 2.96 ± 0.06 | 1.83 ± 0.16 | 4.15 ± 0.21 | 1.24 ± 0.18 |
| *Rheinheimera* | 3.12 ± 0.87 | 2.26 ± 0.48 | 3.43 ± 0.16 | 4.45 ± 0.49 | 0.68 ± 0.08 | 1.29 ± 0.16 |

**Table S13**. Results (*p*-value) of Shapiro-Wilk test for normality and Levene’s test for homogeneity of variances in the relative abundance of antibiotic resistance genes and *intI1* in rainbow trout feces from control, oxytetracycline (OTC)-, and sulfadiazine/trimethoprim (SDZ/TMP)-treated groups before, during, and after antibiotic treatment.

| Period | Day | Test type | Group | *p*-value | | | | | | | | | | | | | | | | |
| --- | --- | --- | --- | --- | --- | --- | --- | --- | --- | --- | --- | --- | --- | --- | --- | --- | --- | --- | --- | --- |
|  |  |  |  | *tetA* | *tetB* | *tetC* | *tetD* | *tetE* | *tetG* | *tetH* | *tetM* | *tetQ* | *tetX* | *tetBP* | *sul1* | *sul2* | *dfrA5* | *dfrA12* | *intI1* |  |
| Pre-treatment | 0 | Normality | Control | 0.87 | 0.95 | 0.73 | 0.76 | 0.53 | 0.66 | 0.66 | 0.48 | 0.65 | 0.37 | 0.11 | 0.05 | 0.14 | 0.73 | 0.44 | 0.96 |  |
|  |  |  | OTC | 0.62 | 0.98 | 0.78 | 0.35 | 0.46 | 0.23 | 0.23 | 0.83 | 0.05 | 0.70 | 0.87 | 0.77 | 0.32 | 0.87 | 0.32 | 0.42 |  |
|  |  |  | SDZ/TMP | 0.94 | 0.98 | 0.32 | 0.12 | 0.91 | 0.87 | 0.87 | 0.05 | 0.74 | 0.32 | 0.73 | 0.95 | 0.81 | 0.12 | 0.37 | 0.95 |  |
|  |  | Homogeneity | All | 0.65 | 0.41 | 0.31 | 0.52 | 0.96 | 0.86 | 0.86 | 0.65 | 0.85 | 0.58 | 0.90 | 0.80 | 0.81 | 0.76 | 0.54 | 0.92 |  |
| Treatment | 1 | Normality | Control | 0.46 | 0.14 | 0.98 | 0.55 | 0.38 | 0.90 | 0.90 | 0.91 | 0.67 | 0.28 | 0.59 | 0.99 | 0.15 | 0.34 | 0.86 | 0.31 |  |
|  |  |  | OTC | 0.77 | 0.30 | 0.37 | 0.05 | 0.74 | 0.69 | 0.69 | 0.82 | 0.29 | 0.36 | 0.66 | 0.91 | 0.05 | 0.55 | 0.81 | 0.81 |  |
|  |  |  | SDZ/TMP | 0.18 | 0.53 | 0.69 | 0.32 | 0.93 | 0.61 | 0.61 | 0.27 | 0.46 | 1.00 | 0.46 | 0.86 | 0.06 | 0.66 | 0.50 | 0.05 |  |
|  |  | Homogeneity | All | 0.80 | 0.86 | 0.48 | 0.66 | 0.47 | 0.43 | 0.43 | 0.92 | 0.39 | 0.36 | 0.70 | 1.00 | 0.79 | 0.26 | 0.84 | 0.99 |  |
|  | 3 | Normality | Control | 0.59 | 0.36 | 0.97 | 0.52 | 0.56 | 0.40 | 0.40 | 0.28 | 0.17 | 0.67 | 0.97 | 0.19 | 0.84 | 0.95 | 0.05 | 0.84 |  |
|  |  |  | OTC | 0.05 | 0.90 | 0.23 | 0.42 | 0.86 | 0.47 | 0.47 | 0.68 | 0.30 | 0.54 | 0.50 | 0.88 | 0.66 | 0.38 | 0.25 | 0.20 |  |
|  |  |  | SDZ/TMP | 0.87 | 0.13 | 0.24 | 0.91 | 0.29 | 0.69 | 0.69 | 0.99 | 0.79 | 0.52 | 0.05 | 0.16 | 0.87 | 0.76 | 0.12 | 0.36 |  |
|  |  | Homogeneity | All | 0.63 | 0.96 | 0.26 | 1.00 | 0.28 | 0.43 | 0.43 | 0.47 | 0.99 | 0.36 | 0.38 | 0.64 | 0.73 | 0.14 | 0.67 | 0.51 |  |
|  | 7 | Normality | Control | 0.66 | 0.58 | 0.71 | 0.05 | 0.87 | 0.25 | 0.25 | 0.68 | 0.05 | 0.16 | 0.28 | 0.42 | 0.59 | 0.14 | 0.67 | 0.81 |  |
|  |  |  | OTC | 0.11 | 0.12 | 0.80 | 0.13 | 0.68 | 0.24 | 0.24 | 0.72 | 0.55 | 0.84 | 0.78 | 0.28 | 0.30 | 0.76 | 0.05 | 0.64 |  |
|  |  |  | SDZ/TMP | 0.06 | 0.16 | 0.48 | 0.42 | 0.21 | 0.62 | 0.62 | 0.43 | 0.89 | 0.74 | 0.92 | 0.05 | 0.11 | 0.38 | 0.69 | 0.59 |  |
|  |  | Homogeneity | All | 0.93 | 0.72 | 0.28 | 0.83 | 0.14 | 0.79 | 0.79 | 0.85 | 0.46 | 0.57 | 0.35 | 0.87 | 0.49 | 0.24 | 0.14 | 0.21 |  |
|  | 10 | Normality | Control | 0.81 | 0.26 | 0.07 | 0.17 | 0.05 | 0.29 | 0.29 | 0.05 | 0.14 | 0.88 | 0.85 | 0.95 | 0.10 | 0.67 | 0.56 | 0.76 |  |
|  |  |  | OTC | 0.05 | 0.69 | 0.09 | 0.80 | 0.72 | 0.05 | 0.05 | 0.20 | 0.87 | 0.62 | 0.05 | 0.27 | 0.49 | 0.91 | 0.49 | 0.05 |  |
|  |  |  | SDZ/TMP | 0.06 | 0.80 | 0.58 | 0.20 | 0.55 | 0.91 | 0.91 | 0.94 | 0.25 | 0.73 | 0.51 | 0.44 | 0.78 | 0.93 | 0.31 | 0.42 |  |
|  |  | Homogeneity | All | 0.84 | 0.46 | 0.83 | 0.62 | 0.34 | 0.66 | 0.66 | 0.55 | 0.75 | 0.29 | 0.80 | 0.67 | 0.74 | 0.13 | 0.53 | 0.66 |  |
| Withdrawal | 11 | Normality | Control | 0.17 | 0.56 | 0.94 | 0.57 | 0.73 | 0.54 | 0.54 | 0.23 | 0.40 | 0.46 | 0.39 | 0.11 | 0.87 | 0.17 | 0.91 | 0.94 |  |
|  |  |  | OTC | 0.44 | 0.77 | 0.87 | 0.05 | 0.41 | 0.32 | 0.32 | 0.05 | 0.71 | 0.55 | 0.55 | 0.84 | 0.39 | 0.98 | 0.13 | 0.55 |  |
|  |  |  | SDZ/TMP | 0.11 | 0.05 | 0.22 | 0.48 | 0.05 | 0.05 | 0.05 | 0.14 | 0.88 | 0.82 | 0.43 | 0.35 | 0.63 | 0.84 | 0.71 | 0.05 |  |
|  |  | Homogeneity | All | 0.51 | 0.48 | 0.19 | 0.73 | 0.35 | 0.31 | 0.31 | 0.54 | 0.83 | 0.50 | 0.92 | 0.37 | 0.24 | 0.43 | 0.12 | 0.53 |  |
|  | 13 | Normality | Control | 0.62 | 0.21 | 0.86 | 0.14 | 0.36 | 0.60 | 0.60 | 0.58 | 0.13 | 0.56 | 0.39 | 0.91 | 0.91 | 0.65 | 0.83 | 0.46 |  |
|  |  |  | OTC | 0.13 | 0.54 | 0.63 | 0.99 | 0.74 | 0.76 | 0.76 | 0.58 | 0.80 | 0.68 | 0.78 | 0.22 | 0.61 | 0.97 | 0.05 | 0.30 |  |
|  |  |  | SDZ/TMP | 0.64 | 0.46 | 0.90 | 0.69 | 0.17 | 0.72 | 0.72 | 0.49 | 0.69 | 0.75 | 0.20 | 0.27 | 0.91 | 0.97 | 0.72 | 0.05 |  |
|  |  | Homogeneity | All | 0.61 | 0.64 | 0.71 | 0.72 | 0.38 | 0.54 | 0.54 | 0.43 | 0.60 | 0.61 | 1.00 | 0.42 | 0.63 | 0.86 | 0.11 | 0.51 |  |
|  | 17 | Normality | Control | 0.78 | 0.15 | 0.58 | 0.72 | 0.81 | 0.77 | 0.77 | 0.52 | 0.84 | 0.41 | 0.90 | 0.36 | 0.41 | 0.81 | 0.32 | 0.32 |  |
|  |  |  | OTC | 0.43 | 0.27 | 0.17 | 0.81 | 0.10 | 0.69 | 0.69 | 0.75 | 0.70 | 0.10 | 0.10 | 0.51 | 0.32 | 0.64 | 0.05 | 0.51 |  |
|  |  |  | SDZ/TMP | 0.60 | 0.92 | 0.59 | 0.05 | 0.40 | 0.21 | 0.21 | 0.48 | 0.79 | 0.47 | 0.49 | 0.44 | 0.18 | 0.19 | 0.05 | 0.45 |  |
|  |  | Homogeneity | All | 0.91 | 0.64 | 0.82 | 0.54 | 0.86 | 0.53 | 0.53 | 0.74 | 0.23 | 0.99 | 0.84 | 0.84 | 0.87 | 0.48 | 0.66 | 0.70 |  |
|  | 24 | Normality | Control | 0.36 | 0.13 | 0.86 | 0.98 | 0.29 | 0.32 | 0.32 | 0.73 | 0.52 | 0.58 | 0.21 | 0.50 | 0.16 | 0.86 | 0.07 | 0.95 |  |
|  |  |  | OTC | 0.75 | 0.13 | 0.20 | 0.42 | 0.41 | 0.05 | 0.05 | 0.16 | 0.05 | 0.14 | 0.78 | 0.14 | 0.64 | 0.27 | 0.38 | 0.32 |  |
|  |  |  | SDZ/TMP | 0.64 | 0.11 | 0.57 | 0.57 | 0.67 | 0.37 | 0.37 | 0.69 | 0.95 | 0.11 | 0.52 | 0.69 | 0.51 | 0.51 | 0.61 | 0.83 |  |
|  |  | Homogeneity | All | 0.54 | 0.51 | 0.57 | 0.68 | 1.00 | 0.76 | 0.76 | 0.40 | 0.56 | 0.83 | 0.89 | 0.41 | 0.88 | 0.81 | 0.67 | 0.53 |  |
|  | 35 | Normality | Control | 0.32 | 0.95 | 0.05 | 0.94 | 0.78 | 0.05 | 0.05 | 0.95 | 0.33 | 0.49 | 0.35 | 0.33 | 0.73 | 0.96 | 0.59 | 1.00 |  |
|  |  |  | OTC | 0.05 | 0.40 | 0.45 | 0.22 | 0.49 | 1.00 | 1.00 | 0.98 | 0.55 | 0.89 | 0.84 | 0.79 | 0.87 | 0.82 | 0.79 | 0.33 |  |
|  |  |  | SDZ/TMP | 0.92 | 0.72 | 0.10 | 0.69 | 0.75 | 0.23 | 0.23 | 0.19 | 0.88 | 0.97 | 0.55 | 0.82 | 0.86 | 0.36 | 0.62 | 0.21 |  |
|  |  | Homogeneity | All | 0.79 | 0.59 | 0.81 | 0.16 | 0.59 | 0.56 | 0.56 | 0.32 | 0.80 | 0.76 | 0.68 | 0.64 | 0.47 | 0.76 | 0.50 | 0.80 |  |

**Table S14**. Mean ± standard deviation of the relative abundance of antibiotic resistance genes and *intI1* in the feces of rainbow trout from the control, oxytetracycline (OTC)-, and sulfadiazine/trimethoprim (SDZ/TMP)-treated groups before, during, and after antibiotic treatment.

| Period | Day | Group | Relative abundance | | | | | | | | | | | | | | | |
| --- | --- | --- | --- | --- | --- | --- | --- | --- | --- | --- | --- | --- | --- | --- | --- | --- | --- | --- |
|  |  |  | *tetA* | *tetB* | *tetC* | *tetD* | *tetE* | *tetG* | *tetH* | *tetM* | *tetQ* | *tetX* | *tetBP* | *sul1* | *sul2* | *dfrA5* | *dfrA12* | *intI1* |
| Pre-treatment | 0 | Control | 1.00 ± 0.75 | 1.00 ± 0.60 | 1.00 ± 0.86 | 1.00 ± 0.76 | 1.00 ± 0.39 | 1.00 ± 0.14 | 1.00 ± 0.51 | 1.00 ± 0.11 | 1.00 ± 0.29 | 1.00 ± 0.39 | 1.00 ± 0.54 | 1.00 ± 0.55 | 1.00 ± 0.54 | 1.00 ± 0.91 | 1.00 ± 0.59 | 1.00 ± 0.37 |
|  |  | OTC | 1.05 ± 0.54 | 1.07 ± 0.27 | 0.88 ± 0.59 | 0.91 ± 0.46 | 1.12 ± 0.40 | 0.88 ± 0.25 | 1.22 ± 0.35 | 0.95 ± 0.26 | 1.30 ± 0.26 | 0.92 ± 0.11 | 1.09 ± 0.55 | 0.84 ± 0.19 | 1.26 ± 0.27 | 1.00 ± 0.41 | 0.96 ± 0.15 | 0.88 ± 0.48 |
|  |  | SDZ/TMP | 1.09 ± 0.34 | 1.02 ± 0.22 | 0.89 ± 0.09 | 1.10 ± 0.24 | 0.84 ± 0.47 | 0.89 ± 0.25 | 0.97 ± 0.60 | 1.05 ± 0.41 | 0.95 ± 0.15 | 0.86 ± 0.58 | 1.02 ± 0.73 | 0.94 ± 0.34 | 0.94 ± 0.26 | 1.06 ± 1.18 | 1.05 ± 0.83 | 1.02 ± 0.33 |
| Treatment | 1 | Control | 1.00 ± 1.01 | 1.00 ± 0.88 | 1.00 ± 0.98 | 1.00 ± 0.81 | 1.00 ± 1.30 | 1.00 ± 1.11 | 1.00 ± 0.70 | 1.00 ± 0.42 | 1.00 ± 0.87 | 1.00 ± 0.71 | 1.00 ± 0.65 | 1.00 ± 0.85 | 1.00 ± 0.38 | 1.00 ± 0.56 | 1.00 ± 0.82 | 1.00 ± 0.46 |
|  |  | OTC | 1.43 ± 0.82 | 1.06 ± 0.48 | 0.86 ± 1.05 | 1.04 ± 0.36 | 1.40 ± 1.41 | 1.06 ± 0.14 | 1.64 ± 0.55 | 1.20 ± 0.36 | 0.81 ± 0.31 | 1.82 ± 0.11 | 0.85 ± 0.58 | 1.18 ± 0.88 | 1.38 ± 0.64 | 0.57 ± 0.12 | 1.05 ± 0.51 | 0.94 ± 0.39 |
|  |  | SDZ/TMP | 1.12 ± 0.55 | 1.16 ± 0.50 | 0.90 ± 0.20 | 0.97 ± 0.45 | 1.01 ± 0.25 | 0.95 ± 0.56 | 1.17 ± 0.62 | 0.87 ± 0.56 | 1.01 ± 0.27 | 0.85 ± 0.08 | 0.86 ± 0.29 | 1.90 ± 0.85 | 2.54 ± 1.11 | 3.81 ± 1.50 | 2.06 ± 0.84 | 0.93 ± 0.48 |
|  | 3 | Control | 1.00 ± 0.50 | 1.00 ± 0.37 | 1.00 ± 0.97 | 1.00 ± 0.37 | 1.00 ± 1.16 | 1.00 ± 1.06 | 1.00 ± 0.31 | 1.00 ± 1.21 | 1.00 ± 0.51 | 1.00 ± 1.00 | 1.00 ± 0.35 | 1.00 ± 0.69 | 1.00 ± 0.98 | 1.00 ± 0.11 | 1.00 ± 1.25 | 1.00 ± 0.10 |
|  |  | OTC | 1.38 ± 0.31 | 0.83 ± 0.46 | 1.09 ± 0.42 | 0.75 ± 0.39 | 4.79 ± 3.43 | 0.93 ± 0.37 | 1.05 ± 0.59 | 0.78 ± 0.23 | 0.85 ± 0.45 | 1.89 ± 0.47 | 0.83 ± 0.52 | 0.60 ± 0.28 | 0.99 ± 0.65 | 0.76 ± 0.36 | 0.73 ± 0.38 | 2.08 ± 0.81 |
|  |  | SDZ/TMP | 0.83 ± 0.17 | 1.17 ± 0.52 | 0.79 ± 0.12 | 0.97 ± 0.38 | 1.22 ± 0.86 | 1.08 ± 0.19 | 0.93 ± 0.14 | 0.75 ± 0.38 | 1.05 ± 0.43 | 0.86 ± 0.20 | 0.89 ± 0.06 | 3.09 ± 1.35 | 1.68 ± 1.23 | 6.29 ± 2.53 | 3.41 ± 0.38 | 1.34 ± 0.30 |
|  | 7 | Control | 1.00 ± 0.67 | 1.00 ± 0.47 | 1.00 ± 0.63 | 1.00 ± 0.44 | 1.00 ± 0.25 | 1.00 ± 0.86 | 1.00 ± 0.27 | 1.00 ± 0.46 | 1.00 ± 0.18 | 1.00 ± 0.49 | 1.00 ± 0.55 | 1.00 ± 0.78 | 1.00 ± 0.88 | 1.00 ± 0.13 | 1.00 ± 0.30 | 1.00 ± 0.25 |
|  |  | OTC | 4.24 ± 0.70 | 1.70 ± 0.79 | 4.34 ± 1.21 | 3.09 ± 0.73 | 21.96 ± 4.62 | 1.68 ± 0.39 | 10.33 ± 3.92 | 1.34 ± 0.55 | 1.70 ± 0.68 | 4.29 ± 1.03 | 1.15 ± 0.99 | 1.64 ± 0.75 | 0.92 ± 0.03 | 1.00 ± 0.51 | 1.34 ± 0.12 | 3.52 ± 0.70 |
|  |  | SDZ/TMP | 0.98 ± 0.48 | 1.40 ± 0.24 | 1.06 ± 0.16 | 0.92 ± 0.32 | 1.36 ± 0.32 | 1.42 ± 0.75 | 0.80 ± 0.44 | 1.03 ± 0.79 | 1.29 ± 0.71 | 1.23 ± 0.51 | 0.81 ± 0.14 | 5.98 ± 1.44 | 1.97 ± 0.95 | 18.16 ± 6.91 | 8.62 ± 2.72 | 29.73 ± 3.62 |
|  | 10 | Control | 1.00 ± 0.51 | 1.00 ± 0.60 | 1.00 ± 1.61 | 1.00 ± 0.68 | 1.00 ± 0.82 | 1.00 ± 1.30 | 1.00 ± 0.83 | 1.00 ± 0.22 | 1.00 ± 0.42 | 1.00 ± 0.53 | 1.00±0.54 | 1.00 ± 0.69 | 1.00 ± 1.40 | 1.00 ± 0.71 | 1.00 ± 0.95 | 1.00 ± 0.35 |
|  |  | OTC | 1.56 ± 0.32 | 1.72 ± 0.07 | 4.22 ± 1.49 | 3.06 ± 0.11 | 12.53 ± 2.06 | 1.16 ± 0.43 | 14.23 ± 3.00 | 2.18 ± 0.57 | 2.61 ± 0.88 | 2.68 ± 0.87 | 0.84 ± 0.47 | 0.91 ± 0.71 | 0.72 ± 0.46 | 1.10 ± 0.60 | 0.87 ± 0.57 | 3.89 ± 1.05 |
|  |  | SDZ/TMP | 1.08 ± 0.69 | 0.82 ± 0.59 | 1.36 ± 0.58 | 0.83 ± 0.39 | 0.73 ± 0.39 | 1.18 ± 0.78 | 0.78 ± 0.23 | 0.93 ± 0.79 | 1.26 ± 0.90 | 1.33 ± 0.04 | 0.99 ± 0.26 | 7.41 ± 1.51 | 3.85 ± 0.78 | 22.80 ± 5.03 | 11.01 ± 2.38 | 43.06 ± 1.40 |
| Withdrawal | 11 | Control | 1.00 ± 0.16 | 1.00 ± 0.42 | 1.00 ± 0.51 | 1.00 ± 0.49 | 1.00 ± 0.18 | 1.00 ± 0.07 | 1.00 ± 0.36 | 1.00 ± 0.79 | 1.00 ± 0.56 | 1.00 ± 0.90 | 1.00 ± 0.66 | 1.00 ± 0.27 | 1.00 ± 0.25 | 1.00 ± 0.45 | 1.00 ± 0.72 | 1.00 ± 0.58 |
|  |  | OTC | 1.51 ± 0.64 | 4.08 ± 1.03 | 9.55 ± 2.65 | 3.20 ± 0.31 | 9.58 ± 1.70 | 1.81 ± 1.34 | 12.31 ± 3.52 | 10.75 ± 2.87 | 2.04 ± 0.30 | 2.42 ± 0.76 | 1.02 ± 0.44 | 1.21 ± 0.89 | 0.97 ± 0.28 | 0.75 ± 0.28 | 1.29 ± 0.37 | 2.23 ± 0.81 |
|  |  | SDZ/TMP | 0.98 ± 0.27 | 1.20 ± 0.42 | 1.34 ± 0.53 | 1.10 ± 0.68 | 1.10 ± 0.47 | 1.56 ± 0.19 | 0.86 ± 0.17 | 1.18 ± 0.14 | 0.94 ± 0.41 | 1.03 ± 0.16 | 1.01 ± 0.64 | 6.49 ± 2.87 | 7.28 ± 1.37 | 2.91 ± 0.94 | 50.33 ± 16.34 | 12.63 ± 4.39 |
|  | 13 | Control | 1.00 ± 0.70 | 1.00 ± 1.31 | 1.00 ± 0.73 | 1.00 ± 0.85 | 1.00 ± 0.29 | 1.00 ± 0.21 | 1.00 ± 0.75 | 1.00 ± 0.23 | 1.00 ± 0.86 | 1.00 ± 0.19 | 1.00 ± 0.47 | 1.00 ± 0.37 | 1.00 ± 0.35 | 1.00 ± 0.29 | 1.00 ± 0.13 | 1.00 ± 0.19 |
|  |  | OTC | 1.24 ± 0.71 | 2.99 ± 0.54 | 2.86 ± 0.77 | 3.01 ± 0.49 | 7.39 ± 1.26 | 1.51 ± 0.60 | 2.97 ± 0.45 | 5.08 ± 0.75 | 2.02 ± 0.57 | 1.53 ± 0.45 | 0.84 ± 0.42 | 1.12 ± 0.67 | 1.01 ± 0.75 | 0.93 ± 0.19 | 1.24 ± 0.18 | 1.86 ± 0.81 |
|  |  | SDZ/TMP | 0.77 ± 0.15 | 0.74 ± 0.40 | 1.07 ± 0.38 | 1.18 ± 0.27 | 1.27 ± 0.55 | 1.39 ± 0.42 | 0.99 ± 0.28 | 1.29 ± 0.27 | 1.23 ± 0.14 | 0.93 ± 0.48 | 0.78 ± 0.49 | 12.82 ± 2.87 | 5.61 ± 0.83 | 2.96 ± 0.22 | 21.66 ± 6.58 | 10.36 ± 3.06 |
|  | 17 | Control | 1.00 ± 0.82 | 1.00 ± 0.50 | 1.00 ± 0.71 | 1.00 ± 0.78 | 1.00 ± 0.43 | 1.00 ± 0.57 | 1.00 ± 0.70 | 1.00 ± 0.51 | 1.00 ± 0.21 | 1.00 ± 0.42 | 1.00 ± 0.51 | 1.00 ± 0.24 | 1.00 ± 0.38 | 1.00 ± 0.77 | 1.00 ± 0.57 | 1.00 ± 0.47 |
|  |  | OTC | 1.19 ± 0.60 | 2.53 ± 0.89 | 1.33 ± 0.45 | 1.43 ± 0.85 | 1.41 ± 0.94 | 1.29 ± 0.30 | 1.07 ± 0.18 | 0.72 ± 0.22 | 1.46 ± 1.01 | 1.35 ± 0.38 | 0.74 ± 0.48 | 1.49 ± 0.40 | 1.43 ± 0.70 | 1.00 ± 0.35 | 1.14 ± 0.53 | 3.63 ± 0.46 |
|  |  | SDZ/TMP | 1.07 ± 0.65 | 1.22 ± 0.21 | 1.15 ± 0.79 | 1.11 ± 0.31 | 0.87 ± 0.63 | 1.11 ± 0.23 | 0.78 ± 0.13 | 0.88 ± 0.40 | 1.21 ± 0.21 | 1.17 ± 0.37 | 0.70 ± 0.27 | 2.40 ± 0.42 | 1.46 ± 0.53 | 1.55 ± 0.30 | 7.73 ± 1.83 | 3.30 ± 0.94 |
|  | 24 | Control | 1.00 ± 0.24 | 1.00 ± 0.21 | 1.00 ± 0.23 | 1.00 ± 0.70 | 1.00 ± 0.26 | 1.00 ± 0.27 | 1.00 ± 0.64 | 1.00 ± 0.57 | 1.00 ± 0.88 | 1.00 ± 0.47 | 1.00 ± 0.45 | 1.00 ± 0.13 | 1.00 ± 0.25 | 1.00 ± 0.81 | 1.00 ± 0.39 | 1.00 ± 0.53 |
|  |  | OTC | 1.15 ± 0.13 | 1.80 ± 0.08 | 1.06 ± 1.00 | 1.11 ± 0.54 | 1.40 ± 0.26 | 1.50 ± 0.84 | 0.74 ± 0.44 | 0.84 ± 0.18 | 1.39 ± 0.75 | 1.42 ± 0.35 | 0.94 ± 0.66 | 1.15 ± 0.14 | 1.15 ± 0.35 | 1.18 ± 0.47 | 0.92 ± 0.38 | 1.24 ± 0.97 |
|  |  | SDZ/TMP | 1.02 ± 0.41 | 1.17 ± 0.75 | 0.88 ± 0.36 | 1.20 ± 0.31 | 0.82 ± 0.25 | 1.39 ± 0.83 | 1.24 ± 0.63 | 1.06 ± 0.89 | 1.12 ± 0.11 | 1.43 ± 0.26 | 0.95 ± 0.56 | 1.09 ± 0.40 | 0.54 ± 0.25 | 0.81 ± 0.70 | 1.62 ± 0.77 | 1.68 ± 0.19 |
|  | 35 | Control | 1.00 ± 0.36 | 1.00 ± 0.65 | 1.00 ± 0.72 | 1.00 ± 0.46 | 1.00 ± 0.08 | 1.00 ± 1.06 | 1.00 ± 0.11 | 1.00 ± 0.66 | 1.00 ± 0.79 | 1.00 ± 0.42 | 1.00 ± 0.65 | 1.00 ± 0.40 | 1.00 ± 0.24 | 1.00 ± 0.39 | 1.00 ± 0.17 | 1.00 ± 0.58 |
|  |  | OTC | 1.10 ± 0.61 | 2.00 ± 0.88 | 0.99 ± 0.43 | 1.17 ± 0.05 | 1.22 ± 0.24 | 0.50 ± 0.16 | 1.27 ± 0.20 | 1.04 ± 0.34 | 1.08 ± 0.46 | 1.05 ± 0.29 | 0.86 ± 0.31 | 1.20 ± 0.78 | 1.23 ± 0.59 | 1.03 ± 0.25 | 0.82 ± 0.51 | 0.80 ± 0.33 |
|  |  | SDZ/TMP | 0.99 ± 0.19 | 0.95 ± 0.25 | 0.92 ± 0.28 | 0.90 ± 0.12 | 0.95 ± 0.15 | 0.59 ± 0.21 | 0.92 ± 0.21 | 0.83 ± 0.15 | 0.98 ± 0.42 | 0.73 ± 0.21 | 0.93 ± 0.28 | 1.65 ± 0.90 | 1.22 ± 0.28 | 1.08 ± 0.56 | 0.73 ± 0.59 | 1.12 ± 0.45 |

| \| **Table S15**. Pearson correlation levels between *intI1* and antibiotic resistance genes in the feces of control, oxytetracycline (OTC)-, and sulfadiazine/trimethoprim (SDZ/TMP)-treated rainbow trout. Level of correlation was assessed using Pearson’s correlation coefficients. \| \| \| \| \| --- \| --- \| --- \| --- \| \| Antimicrobial class \| Antibiotic resistance genes \| Correlation with *intI1* \| \| \| Level of correlation (*r*) \| *p*-value \| \| Tetracycline \| *tetA* \| ᆞ \| 4.E-03 \| \| *tetB* \| ᆞ \| 4.E-02 \| \| *tetC* \| + \| 4.E-05 \| \| *tetD* \| + \| 4.E-06 \| \| *tetE* \| + \| 7.E-04 \| \| *tetG* \| ᆞ \| 7.E-03 \| \| *tetH* \| + \| 1.E-07 \| \| *tetM* \| ᆞ \| 6.E-01 \| \| *tetQ* \| ᆞ \| 2.E-03 \| \| *tetX* \| + \| 5.E-05 \| \| *tetBP* \| No correlation \| 5.E-01 \| \| Sulfonamide \| *sul1* \| ++ \| 2.E-10 \| \| *sul2* \| ++ \| 3.E-11 \| \| Trimethoprim \| *dfrA5* \| ++ \| 1.E-24 \| \| *dfrA12* \| ᆞ \| 3.E-03 \|   ᆞ, weak correlation (0.1≤*r*<0.4); +, moderate correlation (0.4≤*r*<0.7); ++, strong correlation (0.7≤*r*) |
| --- | --- | --- | --- | --- | --- | --- | --- | --- | --- | --- | --- | --- | --- | --- | --- | --- | --- | --- | --- | --- | --- | --- | --- | --- | --- | --- | --- | --- | --- | --- | --- | --- | --- | --- | --- | --- | --- | --- | --- | --- | --- | --- | --- | --- | --- | --- | --- | --- | --- | --- | --- | --- | --- | --- | --- | --- | --- | --- |

| **Table S16**. Correlation with ARGs in differentially abundant genera. The table shows significantly (*p*-value≤0.05) positively associated (+) or not significantly associated (ᆞ) with ARGs in differentially abundant genera in the fecal microbiota of rainbow trout between control and oxytetracycline (OTC)-treated or sulfadiazine/trimethoprim (SDZ/TMP)-treated groups during the 10-day antibiotics treatment. |
| --- |
| \| Bacterial genus \| Correlation with antibiotic resistance related genes \| \| \| \| \| \| \| \| \| \| \| \| \| \| \| \| --- \| --- \| --- \| --- \| --- \| --- \| --- \| --- \| --- \| --- \| --- \| --- \| --- \| --- \| --- \| --- \| \|  \| *tetA* \| *tetB* \| *tetC* \| \| *tetD* \| *tetE* \| *tetG* \| \| *tetH* \| *tetM* \| *tetQ* \| \| *tetX* \| *tetBP* \| \| *Flavobacterium* \| ᆞ \| ᆞ \| + \| \| ᆞ \| ᆞ \| ᆞ \| \| ᆞ \| ᆞ \| ᆞ \| \| ᆞ \| ᆞ \| \| *Glaciecola* \| ᆞ \| ᆞ \| ᆞ \| \| + \| + \| ᆞ \| \| + \| ᆞ \| ᆞ \| \| + \| ᆞ \| \| *Pseudomonas* \| ᆞ \| ᆞ \| + \| \| + \| + \| ᆞ \| \| + \| ᆞ \| ᆞ \| \| + \| ᆞ \| \| *Pseudoxanthomonas* \| ᆞ \| ᆞ \| + \| \| ᆞ \| ᆞ \| ᆞ \| \| ᆞ \| ᆞ \| ᆞ \| \| ᆞ \| ᆞ \| \| *Reyranella* \| ᆞ \| ᆞ \| ᆞ \| \| ᆞ \| ᆞ \| ᆞ \| \| ᆞ \| ᆞ \| ᆞ \| \| ᆞ \| ᆞ \| \| *Streptococcus* \| ᆞ \| ᆞ \| + \| \| + \| + \| ᆞ \| \| + \| + \| ᆞ \| \| + \| ᆞ \| \|  \| *sul1* \| \| \| *sul2* \| \| \| \| *dfrA5* \| \| \| \| *dfrA12* \| \| \| \| \| *Dyadobacter* \| ᆞ \| \| \| ᆞ \| \| \| \| ᆞ \| \| \| \| ᆞ \| \| \| \| \| *Flavobacterium* \| + \| \| \| + \| \| \| \| + \| \| \| \| + \| \| \| \| \| *Methylotenera* \| + \| \| \| + \| \| \| \| + \| \| \| \| + \| \| \| \| \| *Paracoccus* \| ᆞ \| \| \| ᆞ \| \| \| \| ᆞ \| \| \| \| ᆞ \| \| \| \| \| *Pedobacter* \| + \| \| \| + \| \| \| \| + \| \| \| \| + \| \| \| \| \| *Pseudomonas* \| + \| \| \| + \| \| \| \| + \| \| \| \| + \| \| \| \| \| *Pseudoxanthomonas* \| + \| \| \| + \| \| \| \| + \| \| \| \| + \| \| \| \| \| *Reyranella* \| ᆞ \| \| \| ᆞ \| \| \| \| + \| \| \| \| ᆞ \| \| \| \| \| *Rheinheimera* \| + \| \| \| + \| \| \| \| + \| \| \| \| + \| \| \| \| \| *Salinarimonas* \| + \| \| \| + \| \| \| \| + \| \| \| \| + \| \| \| \| |

| **Table S17**. Presence of antibiotic resistance genes in genera. The table shows the harbored (+) or not harbored (-) of ARGs in differently abundant genera in the fecal microbiota of rainbow trout between control and oxytetracycline (OTC)-treated or sulfadiazine/trimethoprim (SDZ/TMP)-treated groups during the 10-day antibiotics treatment. Significant positive correlations (*p*-value≤0.05) between ARGs and microbiome are highlighted with an asterisks. |
| --- |
| \| Bacterial genus \| Presence of antibiotic resistance related genes \| \| \| \| \| \| \| \| \| \| \| \| \| \| \| \| --- \| --- \| --- \| --- \| --- \| --- \| --- \| --- \| --- \| --- \| --- \| --- \| --- \| --- \| --- \| --- \| \|  \| *tetA* \| *tetB* \| *tetC* \| \| *tetD* \| *tetE* \| *tetG* \| \| *tetH* \| *tetM* \| *tetQ* \| \| *tetX* \| *tetBP* \| \| *Flavobacterium* \| - \| - \| - \| \| - \| - \| - \| \| - \| + \| - \| \| + \| - \| \| *Glaciecola* \| - \| - \| - \| \| - \| - \| - \| \| - \| - \| - \| \| - \| - \| \| *Pseudomonas* \| - \| + \| +* \| \| +* \| +* \| + \| \| - \| + \| - \| \| +* \| - \| \| *Pseudoxanthomonas* \| - \| - \| - \| \| - \| + \| - \| \| - \| - \| - \| \| - \| - \| \| *Reyranella* \| - \| - \| - \| \| - \| - \| - \| \| - \| - \| - \| \| - \| - \| \| *Streptococcus* \| - \| + \| +* \| \| - \| - \| - \| \| - \| +* \| - \| \| - \| + \| \|  \| *sul1* \| \| \| *sul2* \| \| \| \| *dfrA5* \| \| \| \| *dfrA12* \| \| \| \| \| *Dyadobacter* \| - \| \| \| - \| \| \| \| - \| \| \| \| - \| \| \| \| \| *Flavobacterium* \| +* \| \| \| +* \| \| \| \| - \| \| \| \| - \| \| \| \| \| *Methylotenera* \| - \| \| \| - \| \| \| \| - \| \| \| \| - \| \| \| \| \| *Paracoccus* \| + \| \| \| + \| \| \| \| - \| \| \| \| - \| \| \| \| \| *Pedobacter* \| - \| \| \| - \| \| \| \| - \| \| \| \| - \| \| \| \| \| *Pseudomonas* \| +* \| \| \| +* \| \| \| \| +* \| \| \| \| +* \| \| \| \| \| *Pseudoxanthomonas* \| +* \| \| \| - \| \| \| \| - \| \| \| \| - \| \| \| \| \| *Reyranella* \| - \| \| \| - \| \| \| \| - \| \| \| \| - \| \| \| \| \| *Rheinheimera* \| +* \| \| \| +* \| \| \| \| - \| \| \| \| - \| \| \| \| \| *Salinarimonas* \| - \| \| \| - \| \| \| \| - \| \| \| \| - \| \| \| \| |

**Reference**

Jun, L. J., Jeong, J. B., Huh, M. D., Chung, J. K., Choi, D. L., Lee, C. H., & Do Jeong, H. (2004). Detection of tetracycline-resistance determinants by multiplex polymerase chain reaction in Edwardsiella tarda isolated from fish farms in Korea. Aquaculture, 240(1-4), 89-100. <https://doi.org/10.1016/j.aquaculture.2004.07.025>.

Mao, D., Yu, S., Rysz, M., Luo, Y., Yang, F., Li, F., ... & Alvarez, P. J. J. (2015). Prevalence and proliferation of antibiotic resistance genes in two municipal wastewater treatment plants.  Water research, 85, 458-466. <https://doi.org/10.1016/j.watres.2015.09.010>.

Srinivasan, V., Nam, H. M., Sawant, A. A., Headrick, S. I., Nguyen, L. T., & Oliver, S. P. (2008). Distribution of tetracycline and streptomycin resistance genes and class 1 integrons in Enterobacteriaceae isolated from dairy and nondairy farm soils. Microbial ecology, 55, 184-193. <https://doi.org/10.1007/s00248-007-9266-6>.

Matter, D., Rossano, A., Limat, S., Vorlet-Fawer, L., Brodard, I., & Perreten, V. (2007). Antimicrobial resistance profile of Actinobacillus pleuropneumoniae and Actinobacillus porcitonsillarum. Veterinary microbiology, 122(1-2), 146-156. <https://doi.org/10.1016/j.vetmic.2007.01.009>.

Tamminen, M., Karkman, A., Lohmus, A., Muziasari, W. I., Takasu, H., Wada, S., ... & Virta, M. (2011). Tetracycline resistance genes persist at aquaculture farms in the absence of selection pressure.  Environmental science & technology, 45 (2), 386-391. <https://doi.org/10.1021/es102725n>.

Munir, M., Wong, K., & Xagoraraki, I. (2011). Release of antibiotic resistant bacteria and genes in the effluent and biosolids of five wastewater utilities in Michigan. Water research, 45(2), 681-693. <https://doi.org/10.1016/j.watres.2010.08.033>.

Aminov, R. I., Garrigues-Jeanjean, N., & Mackie, R. (2001). Molecular ecology of tetracycline resistance: development and validation of primers for detection of tetracycline resistance genes encoding ribosomal protection proteins. Applied and environmental microbiology, 67 (1), 22-32. <https://doi.org/10.1128/AEM.67.1.22-32.2001>.

Ouyang, W. Y., Huang, F. Y., Zhao, Y., Li, H., & Su, J. Q. (2015). Increased levels of antibiotic resistance in urban stream of Jiulongjiang River, China. Applied microbiology and biotechnology, 99, 5697-5707. <https://doi.org/10.1007/s00253-015-6416-5>.

Petrinić, A. (2020). Osjetljivost odabranih komensalnih bakterija iz mikrobiote majčinog mlijeka na antibiotike (Doctoral dissertation, University of Zagreb. Faculty of Food Technology and Biotechnology. Department of Biochemical Engineering. Laboratory for Antibiotic, Enzyme, Probiotic and Starter Cultures Technology). <https://urn.nsk.hr/urn:nbn:hr:159:495945>.

Schwaiger, K., Harms, K., Hölzel, C., Meyer, K., Karl, M., & Bauer, J. (2009). Tetracycline in liquid manure selects for co-occurrence of the resistance genes tet (M) and tet (L) in Enterococcus faecalis. Veterinary microbiology, 139(3-4), 386-392. <https://doi.org/10.1016/j.vetmic.2009.06.005>.

Kim, S. R., Nonaka, L., Oh, M. J., Lavilla-Pitogo, C. R., & Suzuki, S. (2003). Distribution of an oxytetracycline resistance determinant tet (34) among marine bacterial isolates of a Vibrio species. Microbes and environments, 18(2), 74-81. <https://doi.org/10.1264/jsme2.18.74>.

Agersø, Y., & Petersen, A. (2007). The tetracycline resistance determinant Tet 39 and the sulphonamide resistance gene sulII are common among resistant Acinetobacter spp. isolated from integrated fish farms in Thailand. Journal of antimicrobial chemotherapy, 59(1), 23-27. <https://doi.org/10.1093/jac/dkl419>.

Kerrn, M. B., Klemmensen, T., Frimodt-Møller, N., & Espersen, F. (2002). Susceptibility of Danish Escherichia coli strains isolated from urinary tract infections and bacteraemia, and distribution of sul genes conferring sulphonamide resistance.  Journal of antimicrobial chemotherapy, 50 (4), 513-516. <https://doi.org/10.1093/jac/dkf164>.

Xiong, W., Sun, Y., Zhang, T., Ding, X., Li, Y., Wang, M., & Zeng, Z. (2015). Antibiotics, antibiotic resistance genes, and bacterial community composition in fresh water aquaculture environment in China. Microbial ecology, 70, 425-432. <https://doi.org/10.1007/s00248-015-0583-x>.

Pei, R., Kim, S. C., Carlson, K. H., & Pruden, A. (2006). Effect of river landscape on the sediment concentrations of antibiotics and corresponding antibiotic resistance genes (ARG). Water research, 40(12), 2427-2435. <https://doi.org/10.1016/j.watres.2006.04.017>.

Grape, M., Motakefi, A., Pavuluri, S., & Kahlmeter, G. (2007). Standard and real-time multiplex PCR methods for detection of trimethoprim resistance dfr genes in large collections of bacteria.  Clinical Microbiology and Infection, 13 (11), 1112-1118. <https://doi.org/10.1111/j.1469-0691.2007.01807.x>.

Kim, A., Lim, Y., Kim, N., Luan Nguyen, T., Roh, H. J., Park, C. I., ... & Smith, P. (2018). A comparison of genotypic and phenotypic methods for analyzing the susceptibility to sulfamethoxazole and trimethoprim in Edwardsiella piscicida. Microbial Drug Resistance, 24(8), 1226-1235. https://doi.org/10.1089/mdr.2017.0137.

Okoh, A. I., & Igbinosa, E. O. (2010). Antibiotic susceptibility profiles of some Vibrio strains isolated from wastewater final effluents in a rural community of the Eastern Cape Province of South Africa. BMC microbiology, 10, 1-6. <https://doi.org/10.1186/1471-2180-10-143>.

Amador, P., Fernandes, R., Prudêncio, C., & Duarte, I. (2019). Prevalence of antibiotic resistance genes in multidrug-resistant Enterobacteriaceae on Portuguese livestock manure. Antibiotics, 8(1), 23. <https://doi.org/10.3390/antibiotics8010023>.

Ng, L. K., Martin, I., Alfa, M., & Mulvey, M. (2001). Multiplex PCR for the detection of tetracycline resistant genes.  Molecular and cellular probes, 15 (4), 209-215. <https://doi.org/10.1006/mcpr.2001.0363>.

Barraud, O., Baclet, M. C., Denis, F., & Ploy, M. C. (2010). Quantitative multiplex real-time PCR for detecting class 1, 2 and d3 integrons.  Journal of antimicrobial chemotherapy, 65 (8), 1642-1645. <https://doi.org/10.1093/jac/dkq167>.

Muyzer, G., De Waal, E. C., & Uitterlinden, A. (1993). Profiling of complex microbial populations by denaturing gradient gel electrophoresis analysis of polymerase chain reaction-amplified genes coding for 16S rRNA.  Applied and environmental microbiology, 59 (3), 695-700. <https://doi.org/10.1128/aem.59.3.695-700.1993>.
